# Investigating the Chemical Ordering in Quaternary Clathrate Ba_8_Al_*x*_Ga_16–*x*_Ge_30_

**DOI:** 10.1021/acs.inorgchem.1c01932

**Published:** 2021-11-03

**Authors:** Yifei Zhang, Joakim Brorsson, Takashi Kamiyama, Takashi Saito, Paul Erhart, Anders E. C. Palmqvist

**Affiliations:** † Department of Chemistry and Chemical Engineering, Chalmers University of Technology, 41296 Gothenburg, Sweden; ‡ Institute of Materials Structure Science, KEK, Tokai, Ibaraki319-1106, Japan; ¶ SOKENDAI (The Graduate University for Advanced Studies), Tokai-mura, Naka-gun, Ibaraki 319-1106, Japan; § Department of Physics, Chalmers University of Technology, 41296 Gothenburg, Sweden

## Abstract

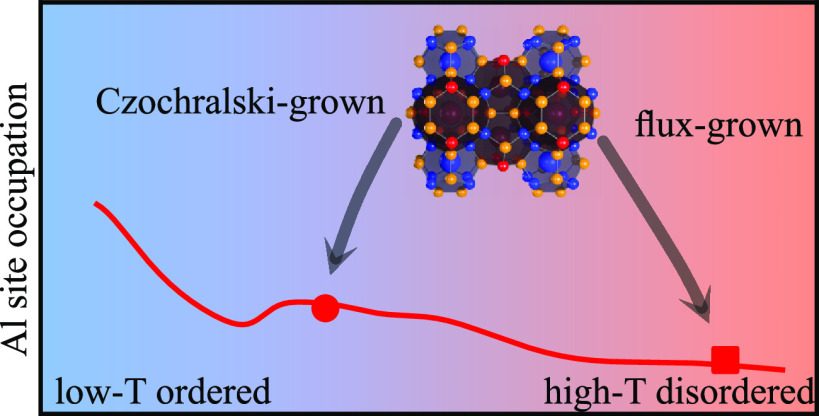

Recently, there has been an increased interest in quaternary clathrate systems as promising thermoelectric materials. Because of their increased complexity, however, the chemical ordering in the host framework of quaternary clathrates has not yet been comprehensively analyzed. Here, we have synthesized a prototypical quaternary type-I clathrate Ba_8_Al_*x*_Ga_16–*x*_Ge_30_ by Czochralski and flux methods, and we employed a combination of X-ray and neutron diffraction along with atomic scale simulations to investigate chemical ordering in this material. We show that the site occupancy factors of trivalent elements at the 6*c* site differ, depending on the synthesis method, which can be attributed to the level of equilibration. The flux-grown samples are consistent with the simulated high-temperature disordered configuration, while the degree of ordering for the Czochralski sample lies between the ground state and the high-temperature state. Moreover, we demonstrate that the atomic displacement parameters of the Ba atoms in the larger tetrakaidecahedral cages are related to chemical ordering. Specifically, Ba atoms are either displaced toward the periphery or localized at the cage centers. Consequently, this study reveals key relationships between the chemical ordering in the quaternary clathrates Ba_8_Al_*x*_Ga_16–*x*_Ge_30_ and the structural properties, thereby offering new perspectives on designing these materials and optimizing their thermoelectric properties.

## Abstract 2

The chemical ordering of quaternary clathrate Ba_8_Al_*x*_Ga_16−*x*_Ge_30_ is determined, showing that the Al occupation at the 6*c* site differs explicitly, depending on the synthesis methods. It is further corroborated by the theoretical calculations that the flux-grown samples are consistent with the simulated high-temperature disordered configuration, while the degree of ordering for the Czochralski sample lies between the ground state and the high-temperature state.

## Introduction

**Figure 1:**
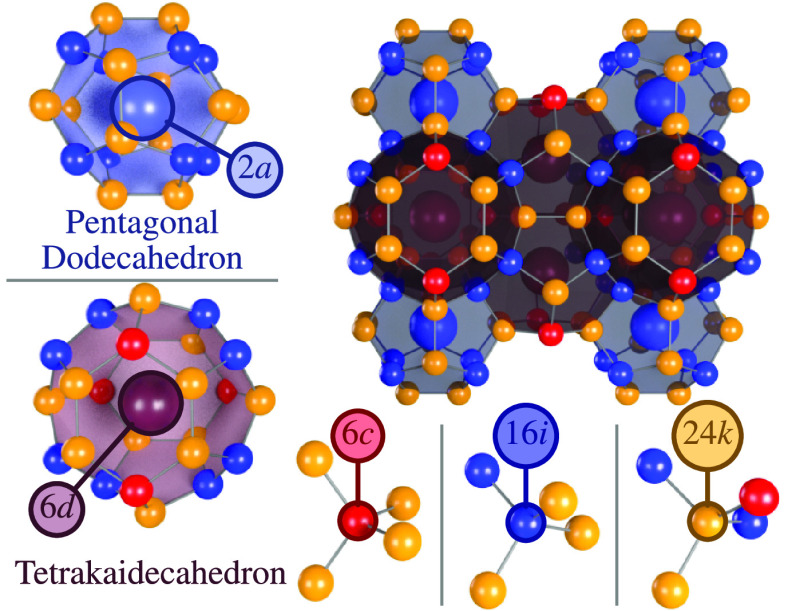
Crystal structure of type-I clathrate (space group *Pm*3̅*n*). The host atoms occupy Wyckoff sites 6*c*, 16*i*, and 24*k*, forming small pentagonal dodecahedral and large tetrakaidecahedral cages, while the guest atoms are located at the cage centers, corresponding to 2*a* and 6*d* sites.

The thermoelectric effect enables a direct conversion between a temperature gradient and an electrical potential and can be used in applications such as power generation, waste heat recovery, and, reversely, in active cooling.^1^ One promising group of thermoelectric materials is type-I inorganic clathrates. They can be regarded as realizations of the phonon-glass electron-crystal concept, combining relatively large electrical conductivity (“electron crystal”) with very low thermal conductivity (“phonon glass”).^2^ Type-I clathrates have the general composition Z_8_A_16_B_30_, where Z refers to the so-called guest element, typically an alkaline-earth metal, while A and B stand for the host lattice and usually come from groups 13 and 14 of the periodic table.^3^ As shown in Figure 1, host atoms are located at Wyckoff sites 6*c*, 16*i*, and 24*k* and connected with covalent bonds, forming two different types of cages–small pentagonal dodecahedral and large tetrakaidecahedral cages. The guest atoms, meanwhile, are located at the cage centers, which correspond to Wyckoff positions 2*a* and 6*d*, respectively.

Inorganic type-I clathrates are characterized as Zintl phases, which means that the guest atoms do not directly participate in the bonding, but rather donate valence electrons to the bonds between the host atoms.^4^ In a typical type-I clathrate compound such as Ba_8_Ga_16_Ge_30_, the total number of electrons donated by each Ba atom (nominally +2) is balanced by the relative electron deficiency of each Ga atom (nominally −1) needed for tetravalent bonding configurations of all host elements in the unit cell. Thus, the material should behave as an intrinsic semiconductor. In practice, however, the real composition of synthesized samples usually deviates somewhat from the ideal stoichiometry and may also contain defects such as vacancies. As a result, the samples typically display *n*-type or *p*-type semiconducting or even metallic behavior. Even with the same nominal composition, the thermoelectric properties of clathrates may differ between samples. This can be due to chemical ordering, which is known to affect the band structure.^5−8^ More specifically, the chemical ordering in clathrates refers to the occupation of the host sites 6*c*, 16*i*, and 24*k* by chemically distinct atoms (Al, Ga, and Ge). As mentioned above, the host elements occupy three different positions (6*c*, 16*i*, and 24*k* in Wyckoff notation), but are not randomly distributed over these sites. For instance, the trivalent elements rather preferably occupy the 6*c* site, because the direct bonding between trivalent elements is energetically unfavorable.^6,7,9^ The degree of chemical ordering can also vary, depending on the synthesis method used to prepare the material. For example, Christensen et al.^9^ have reported that, in a Ba_8_Al_16_Ge_30_ single crystal grown by the Czochralski method, the 6*c* and 24*k* sites are almost 100% occupied by Al and Ge, respectively. Conversely, they found that the Al occupation at the 6*c* site was only 55%, while a totally random distribution was observed at the 24*k* site for their flux-grown samples. The chemical ordering in the host framework also has an impact on the guest atoms. A neutron diffraction study of Ba_8_Al_*x*_Si_46–*x*_ revealed that the direction of the anisotropic atomic displacement for the Ba atoms at the 6*d* site is influenced by the site occupation of Al, suggesting that the thermal conductivity could also be affected.^10^

Quaternary clathrates have received increased attention in recent years and unconventional elements, such as transition metals or even group 15 elements, have been introduced into the host framework.^11−15^ However, studies of the chemical ordering in quaternary clathrates are rare, probably because they typically contain elements that are close in the periodic table and, hence, are difficult to distinguish by X-ray techniques.^15,16^ Notwithstanding, Puspita et al.^17^ have reported a structural study of Ba_8_Al_*x*_Ga_16–*x*_Ge_30_ (*x* ≥ 8) using powder X-ray and neutron diffraction. Although the chemical ordering they observed agrees qualitatively with empirical rules, a systematic investigation of the system is still lacking.

In this study, samples from the quaternary clathrate series Ba_8_Al_*x*_Ga_16–*x*_Ge_30_ (*x* ≤ 8) were synthesized by two methods, Czochralski pulling and the Ga flux growth. The samples thus obtained were subsequently investigated by a combination of X-ray and neutron diffraction, and the measured site occupation factors (SOFs) were compared to complementary atomic scale simulations.

## Results and Discussion

Accurate determination of the composition of inorganic clathrates has proven challenging, since these compounds are usually composed of neighboring elements whose characteristic peaks overlap in the X-ray fluorescence (XRF) spectrum or have similar X-ray scattering cross sections.^16^ Quaternary Ba_8_Al_*x*_Ga_16–*x*_Ge_30_ compounds are even more problematic, since the material contains only 3–5 wt % of the lightest element (Al). In order to study the chemical ordering, the local chemical environment of each element must be investigated. While this can, in principle, be done by solid-state nuclear magnetic resonance (NMR), this approach cannot distinguish the chemical environments of 16*i* and 24*k* sites.^14^ In the present study, we therefore employ X-ray and neutron diffraction to study the chemical ordering in Ba_8_Al_*x*_Ga_16–*x*_Ge_30_, and compare the results with atomic scale simulations, using the methodology outlined below.

Samples are first characterized by refinement of single-crystal X-ray diffraction (XRD). Since Ga and Ge have similar X-ray cross sections, only the Al SOFs are refined. All sites are assumed to be 100% occupied and to have isotropic atomic displacement parameters (ADPs), except for the Ba atoms at the 6*d* site, which possess anisotropic ADPs. The composition obtained from the structure refinement is compared with that from X-ray fluorescence (XRF) analysis. Hereafter, we refer to our samples by a code such as F-Al0.0 or C-Al5.2, where the first letter indicates the growth method (F = flux-grown; C = Czochralski-pulled) and the number indicates the Al content, according to XRD.

While we do find evidence of vacancies for some compositions (such as F-Al0.0, as described below), we cannot establish the existence of vacancies in the other samples (C-Al5.2, F-Al6.3, F-Al6.7, F-Al7.4, and F-Al8.8) when analyzing the electrical transport properties. A detailed analysis will be published separately.

Next, the SOFs of Ga and Ge are examined by neutron diffraction. The structure refinement is similar to the one described above, but with a few constraints: The SOFs of Al is kept fixed and the number of Ge atoms is set to 30 per unit cell. The physical properties (lattice parameter, ADP, and atomic position) obtained from neutron diffraction are comparable to those obtained from XRD, meaning that both structure refinements are reasonable. Although a few impurity peaks are visible in the powder neutron diffraction patterns, their intensities are low, and since these are not observed when using laboratory powder XRD, they are excluded from the refinement.

Finally, the chemical ordering determined by the experiments is compared to the results from a combination of density functional theory (DFT), alloy cluster expansion (CE), and Monte Carlo (MC) simulations.

### Elemental Composition

While the absolute amount of Al in the prepared materials is difficult to determine by XRF, the Ga, Ge, and Ba content can be measured (Table 1). It is found that the Ge content is either close to or slightly higher than 30 atoms per unit cell for low-Al samples (*x* < 7). Since the flux-grown samples are synthesized in an excess of Ga, it can be argued that these samples should be Ga-rich and Ge-deficient. Yet, we find that the composition of the as-grown single crystals can, in fact, be tuned by varying the starting composition. In previous studies, the starting composition was usually Ba:Ga:Ge = 8:64:30,^18,19^ while for our F-Al6.3 and F-Al6.7 samples, the Ba:Al:Ga:Ge ratios are 8:10:28:30 and 8:12:28:30, respectively. By using a smaller Ga flux, it is possible to increase the Ge content and thereby produce single crystals with almost stoichiometric compositions (Ba_8_Al_*x*_Ga_16–*x*_Ge_30_). On the other hand, a double flux,^20^ consisting of excess Al and Ga, was used to prepare samples F-Al7.4 and F-Al8.8. As a result, the crystals are Ge-deficient, containing only 29.3 and 29.1 Ge atoms per unit cell, respectively.

**Table 1:** Elemental Compositions of Ba_8_Al_*x*_Ga_16–*x*_Ge_30_ Obtained from XRF^a^ and XRD^b^^,^^c^

	Starting Composition			Composition from XRF				Composition from XRD	
sample	Al	Ga	Ge	Al	Ga	Ge	Ga + Ge	Al	Ga + Ge
F-Al0.0	0	28	30	0	15.4	30.0	45.4	0	45.2(3)
C-Al5.2	4	12	30	4.0	11.5	30.5	42.0	5.2(8)	40.8(8)
F-Al6.3	10	28	30	4.5	11.5	30.0	41.5	6.3(8)	39.7(8)
F-Al6.7	12	28	30	4.7	11.0	30.3	41.3	6.7(8)	39.3(8)
F-Al7.4	16	28	30	7.0	9.7	29.3	39.0	7.4(8)	38.6(8)
F-Al8.8	20	28	30	8.5	8.4	29.1	37.5	8.8(8)	37.2(8)

a The Al content obtained from XRF is calculated as Al = 46-(Ga + Ge).

b Values in parentheses refer to standard deviations.

c Composition is normalized to 8 Ba atoms per unit cell. XRF spectra are in the Supporting Information.

**Figure 2:**
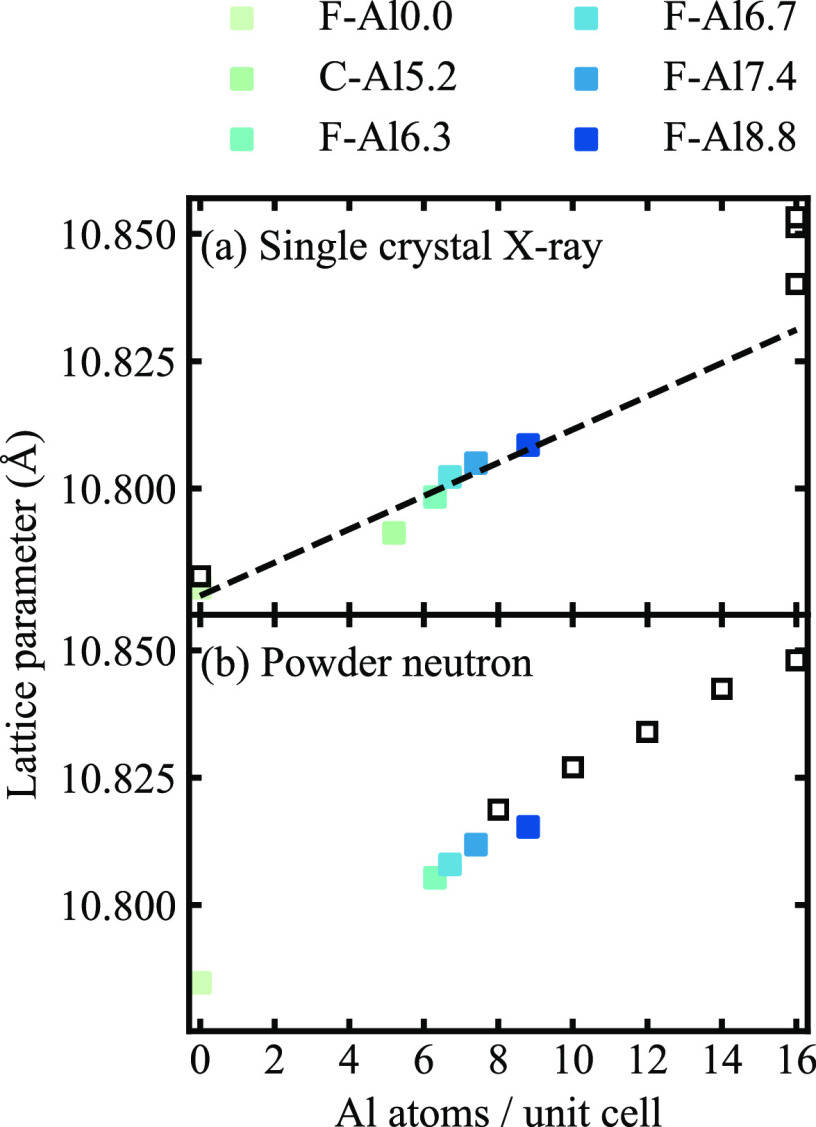
Lattice parameter at 300 K versus Al content from (a) single-crystal X-ray diffraction and (b) powder neutron diffraction experiments. Data obtained in this work are shown as solid colored squares, while reference data from the literature is shown by black empty squares. [Reproduced from refs (9) (Copyright 2007, American Chemical Society, Washington, DC), (17) (Copyright 2006, American Chemical Society, Washington, DC), (19) (Copyright 2018, Elsevier), and (21) (Copyright 2019, IEEE).]

As more Al is added to the starting composition, the amount of Al in the obtained crystals increases correspondingly, as is confirmed by both XRF and XRD. Although the Al content is lower than the starting composition, this does not mean that the solubility limit of Al in Ba_8_Al_*x*_Ga_16–*x*_Ge_30_ has been reached. Rather, the reason is that a certain amount of Al also dissolves in the Ga flux. This conclusion is further supported by our measurements of the lattice parameter of Ba_8_Al_*x*_Ga_16–*x*_Ge_30_ (Figure 2). The lattice parameter increases linearly with the number of Al atoms (10.7789 Å + 0.00326 Å*x*), consistent with the fact that Ba_8_Al_16_Ge_30_ has a larger lattice parameter than Ba_8_Ga_16_Ge_30_. The unit cells of our materials are smaller than those previously reported for Ba_8_Al_*x*_Ga_16–*x*_Ge_30_ (8 ≤ *x* ≤ 14; see Figure 2b), as expected due to the lower Al content.

The compositions determined by XRD are comparable to those from XRF (Table 1). Still, there are variations, depending on the model used to describe the guest atoms. Specifically, the Ba atoms in the large cages can be modeled as being either on-center or off-center; the goodness-of-fit is almost identical for both models, but the latter gives a lower Al content. For instance, in the case of sample C-Al5.2, the Al content is 5.2(8) for the on-center model, but only 4.1(8) for the off-center model. Since we lack a reliable and independent measure of the Al content, we are, unfortunately, unable to say which of them is the more accurate. In order to better compare our results with previous studies, we have chosen to use the on-center model.

### Chemical Ordering of Host Sites

Even though the Ba_8_Al_*x*_Ga_16–*x*_Ge_30_ samples can be expected to exhibit both substitutional disorder and positional disorder, reasonably low goodness-of-fit (GOF) values are obtained (see the Supporting Information). The consistency of the lattice parameters and ADPs obtained from X-ray and neutron diffraction (Figure S2 in the Supporting Information) also lends support to the proposed methodology and structure model.

**Figure 3:**
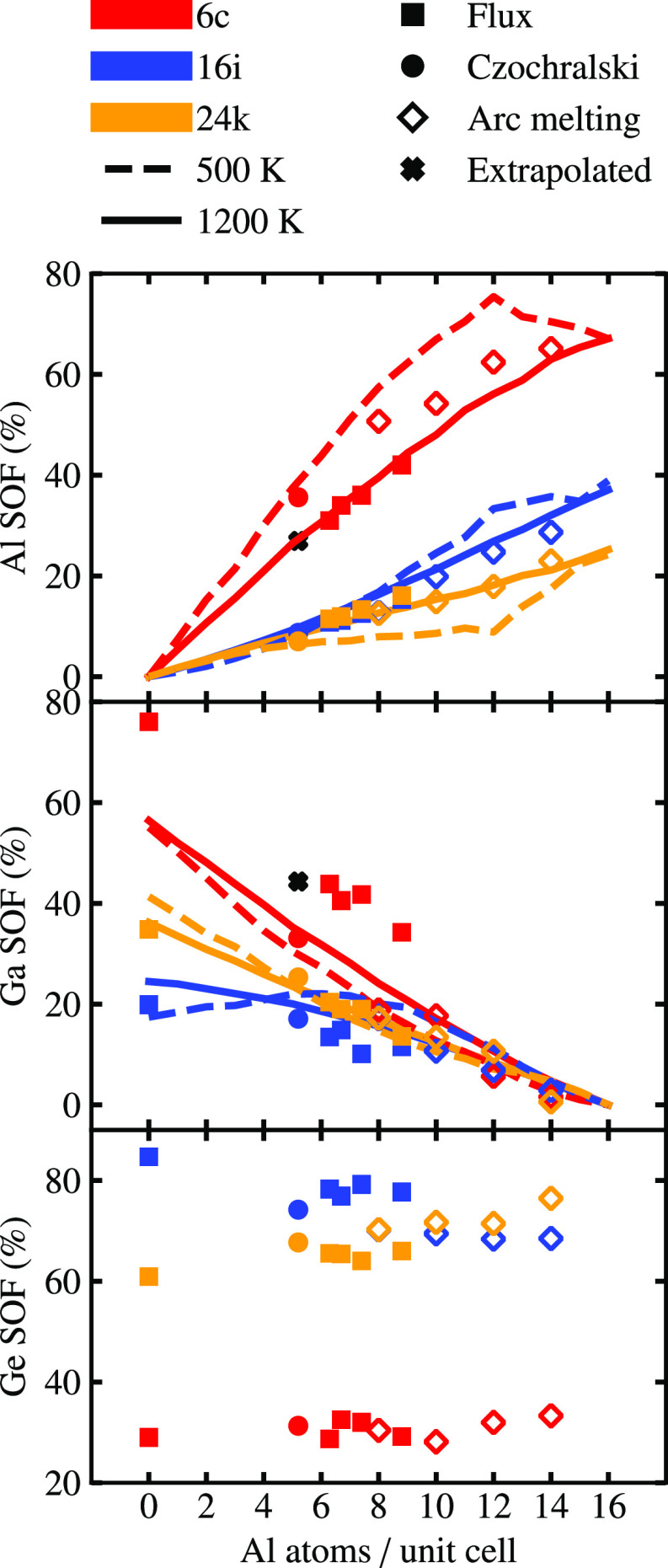
SOFs of the host elements (Al, Ga, and Ge) at the 6*c* (red), 16*i* (blue), and 24*k* (orange) sites from experiment ((●) Czochralski, (■) flux-grown, and (◇) arc-melted samples^17^) and simulation ((**− – –**) 500 K and (**—**) 1200 K). Black crosses (**+**) indicate SOFs at *x* = 5.2 obtained by extrapolation of the data from flux-grown samples. [Reproduced from ref (17). Copyright 2019, IEEE.]

**Figure 4:**
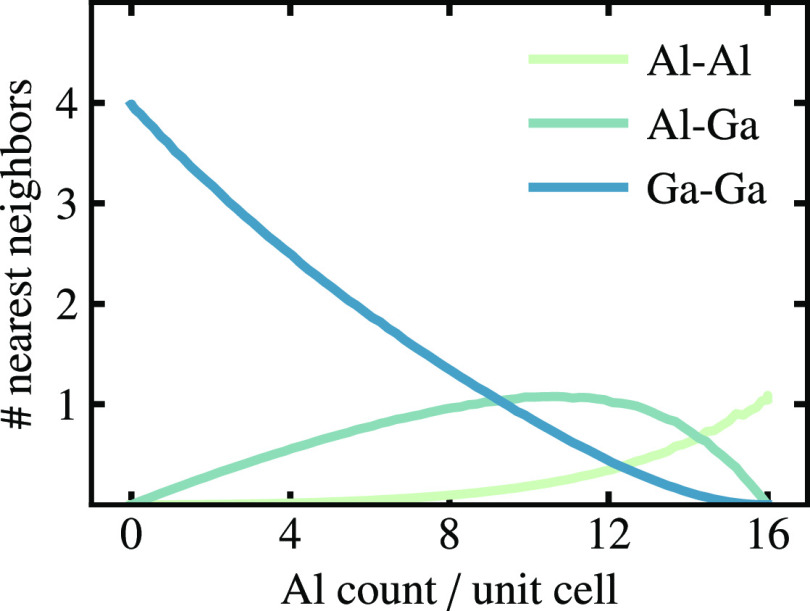
Number of Al–Al (green), Al–Ga (blue-green), and Ga–Ga (blue) nearest neighbors per unit cell versus composition extracted from MC simulations at 300 K.

As shown in Figure 3, the Ge SOF at the 6*c* site remains almost constant when *x* increases from 0 to 8.8, giving values of 29.0(18)% and 29.2(14)%, respectively, similar to the literature data^17^ (33.3% at *x* = 14). Considering the measurement accuracy, this implies that Ge at the 6*c* site is not substituted by Al at all. In contrast, the Ge SOFs at the other two host sites exhibit large variations: at the 16*i* site, it decreases from 84.7(10)% (F-Al0.0) to 77.7(7)% (F-Al8.8), and at the 24*k* site, it increases from 60.9(6)% to 66.0(4)%. When a wider composition range is considered, including literature data for arc-melted Ba_8_Al_*x*_Ga_16–*x*_Ge_30_ (8 ≤ *x* ≤ 14),^17^ it is confirmed that the Ge SOFs at the 16*i* and 24*k* sites decrease and increase, respectively, with Al content. This can, qualitatively, be attributed to the fact that the material has a tendency to avoid direct bonding between trivalent elements. As shown in Figure 4, Al–Al is the least favorable bond and simulations predict it to be almost absent in Ba_8_Al_*x*_Ga_16–*x*_Ge_30_, while Ga–Ga bonds can be tolerated to some extent. With increasing Al content, 6*c* sites are occupied first, followed by 16*i*, which are preferable over 24*k* sites, because the latter are directly connected to 6*c* sites. Further support for this conclusion can be found in the literature; for instance, it has been reported that in Ba_8_Al_16_Ge_30_, synthesized by the Czochralski method, the 6*c* and 24*k* sites are almost 100% occupied by Al and Ge, respectively.^9^

The trivalent elements Al and Ga are found on all the host sites, but preferably occupy the 6*c* site, which is consistent with the empirical rules proposed by Christensen et al.^9^ as well as electronic structure-based lattice simulations.^22^ In addition, Al is almost equally distributed between the 16*i* and 24*k* sites, while Ga shows a higher preference for the 24*k* site. These results agree with the conclusion that the material, as mentioned above, has a strong tendency to avoid Al–Al bonds, and, to some extent, also Al–Ga bonds. Interestingly, the occupation of the 6*c* site differs between samples synthesized by different methods as the Al (Ga) occupation at the 6*c* site in the Czochralski and arc-melted^17^ samples are systematically higher (lower) than those synthesized via the flux method (Figure 3). For the 16*i* and 24*k* sites, the difference between flux and Czochralski-grown samples are smaller and within the standard deviation of structure refinement results.

### Degree of Chemical Ordering

Controlling the chemical ordering enables tuning the electrical properties, which is a crucial capability in the semiconductor field in general^5^ and for thermoelectric materials in particular. In terms of thermodynamics, entropy has a larger impact at high temperatures, where it may drive materials that are ordered at low temperatures to form disordered structures. The degree of chemical ordering is reflected not only in the SOFs but also short- and long-range order parameters, which can provide a chemically more intuitive picture as they relate more directly to the atomic scale interactions that drive order. In principle, diffraction techniques enable the determination of long-range order parameters while short-range order can be probed, for instance, via the extended X-ray fine structure atomic pair distribution function.^5,23−25^ In the case of clathrates, long-range chemical order usually does not emerge. On the other hand, the analysis of short-range order is very complicated, because it involves three different species, with poor contrast in terms of their X-ray fine structure. Here, we therefore resort to atomic-scale simulations, which can predict the thermodynamically stable configuration at a given temperature, and have been shown to provide reliable estimates of the SOFs for ternary clathrates.^7^

**Figure 5:**
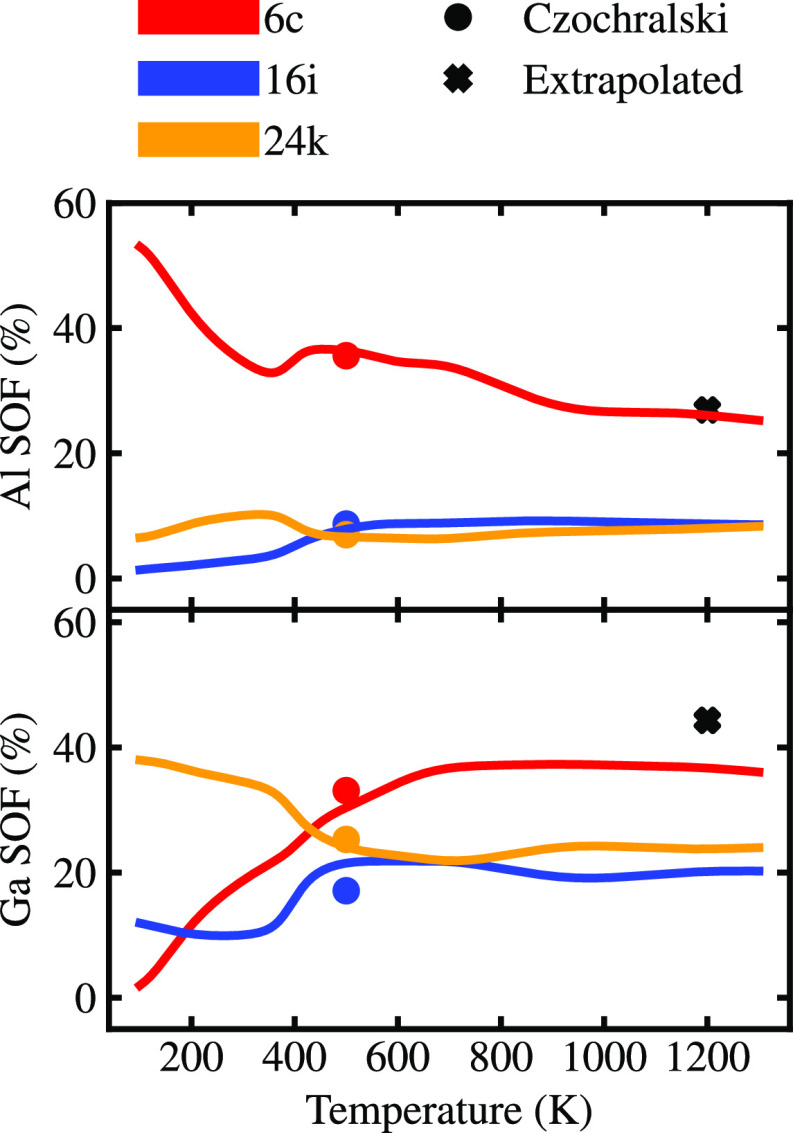
Variation of the simulated Al and Ga SOFs with temperature at the 6*c* (red), 16*i* (blue), and 24*k* (orange) Wyckoff sites for Ba_8_Al_*x*_Ga_16–*x*_Ge_30_ (*x* = 5), together with experimental data points for the Czochralski sample ((●)) and extrapolated data from flux-grown samples (**+**).

In order to check the validity of our experimental observations, as well as characterize the degree of chemical ordering, we have applied a similar approach to study quaternary clathrates, building on our recent analysis of order–disorder transitions in these systems.^8^ The existence of this transformation is evident from the temperature variations of the Al and Ga SOFs, respectively, in Ba_8_Al_*x*_Ga_16–*x*_Ge_30_ with *x* = 5 (*x* = 4, 6, 8) (see Figure 5, as well as Figure S3a in the Supporting Information). The SOFs for both elements change dramatically at the order–disorder transition temperature of ∼400 K and then change gradually until 1200 K, which is close to the melting point of Ba_8_Al_16_Ge_30_ and Ba_8_Ga_16_Ge_30_. By comparing the calculated SOFs with our experimental data (Figure 5), it is found that our Czochralski sample is consistent with the calculated configuration at 500 K, while the extrapolated SOFs of the flux-grown sample correspond better to the high-temperature configuration. When this analysis is extended over a wider composition range (0 ≤ *x* ≤ 14; Figure 3), it leads to the conclusion that the flux-grown samples are consistent with high-temperature configurations while the SOFs for the arc-melted and Czochralski samples represent a state of order that is intermediate between the calculations for the high-temperature and ground states. With respect to the degree of chemical ordering, the flux-grown samples are, in other words, more disordered, compared to the others.

Therefore, the degree of chemical ordering in Ba_8_Al_*x*_Ga_16–*x*_Ge_30_ can, in fact, be influenced by tuning the experimental conditions, e.g., the reaction temperature, cooling rate, and chemical environment (for example, extra Ga flux). As shown in Figure S3b in the Supporting Information, however, most experimental data fall within the standard deviation of the calculated SOFs at 700 K. Even though the significantly higher Ga occupation at the 6*c* sites observed for flux-grown samples could simply be attributed to the excess Ga used in the synthesis, our results suggest that it is a direct consequence of the intrinsic ordering tendency of this system. The degree of ordering could also explain the inconsistencies in electrical transport properties that have previously been observed,^26^ even for samples with the same nominal compositions.

### Atomic Displacement Parameter of Guest Atoms

Inorganic clathrates are known for their intrinsically low thermal conductivity. Although they possess a crystalline structure, the lattice thermal conductivity is close to the amorphous limit. The origin of the low thermal conductivity has been debated, but mainly attributed to the interaction between the vibration modes of the guest atoms and of the host framework. Because the guests at the 6*d* sites are loosely bound within oversized cages, they exhibit large anisotropic ADPs and have sometimes been described as atomic “rattlers”.^9,19^

**Figure 6:**
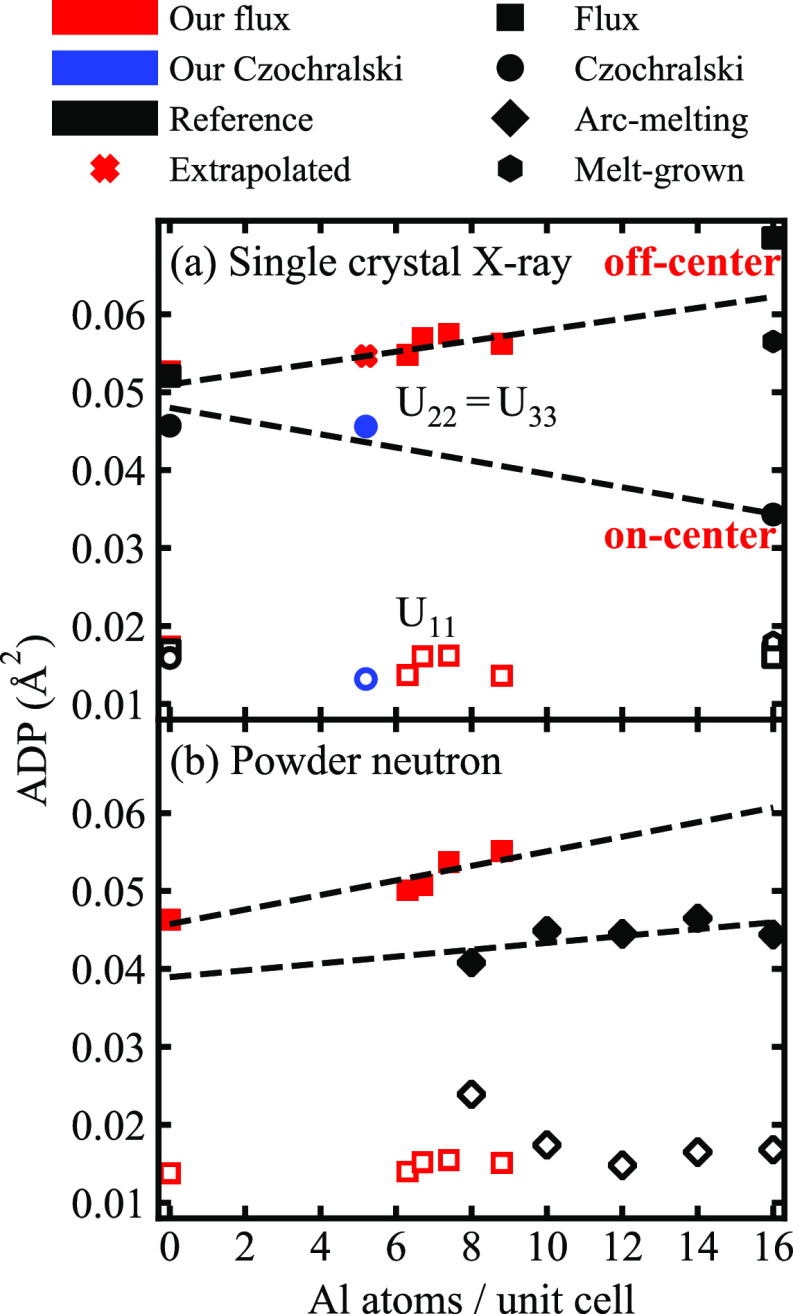
Anisotropic ADPs *U*_22_ = *U*_33_ (solid data markers) and *U*_11_ (open data markers) for the Ba atoms at the 6*d* site versus the Al content in Ba_8_Al_*x*_Ga_16–*x*_Ge_30_ for our flux-grown (red squares) and Czochralski-pulled (blue circles) samples, together with reference samples including (black squares) flux-grown, (black circles) Czochralski-pulled, (black hexagons, M) melt-grown, and (black diamonds) arc-melting samples (see Figure 2 for references). Dashed lines are linear fits of the ADPs for samples grown using flux-growth (solid red squares), Czochralski (solid circles), and arc-melting (solid black diamonds). The cross (+) represents the extrapolated values at *x* = 5.2. [Reproduced from refs (9) (Copyright 2007, American Chemical Society, Washington, DC), (17) (Copyright 2006, American Chemical Society, Washington, DC), (19) (Copyright 2018, Elsevier), and (21) (Copyright 2019, IEEE).]

Previously, the displacement parameter of the guest atoms was thought to be dependent mainly on the cage size, meaning that a smaller guest atom in a larger cage should exhibit a higher ADP.^27^ However, it has later been found that the rattling nature of guest atoms is more complex, and that the host atom distribution also plays a role.^10^ As a result, the reported values on *U*_22_ for Ba_8_Al_16_Ge_30_, for instance, vary over a wide range (Figure 6). Note that a smaller ADP means that the guest atom vibrates more closely at the cage center (on-center), while a larger value indicates that it undergoes larger thermal motion and could possibly be better described by an off-center model. In contrast, Ba_8_Ga_16_Ge_30_ shows quite uniform ADPs. Differences in the displacement parameter are also observed for quaternary Ba_8_Al_*x*_Ga_16–*x*_Ge_30_, depending on the synthesis method (Figure 6). Specifically, *U*_22_ increases with Al content for the flux samples, indicating that the Ba atoms are situated closer to the cage peripheries. The value for sample C-Al5.2, on the other hand, is significantly smaller.

It is desirable to identify the reason for the different displacement parameters. The lattice parameter, as well as the bond distances between host and guest atoms, increase linearly with Al content (see Figure 2, as well as Figure S4 in the Supporting Information), which means that the volume of the large tetrakaidecahedral cage also increases (cage volume is based on the distance between the guest and host atoms centers). If the cage size is the determining factor, one would, accordingly, expect that the displacement parameter for sample C-Al5.2 should be comparable to the value extrapolated from the flux-grown samples, indicated by the black cross, or at least higher than F-Al0.0. Yet, this is not the case. A comparison between our results and those reported by Puspita et al.^17^ (8 ≤ *x* ≤ 14) provides further evidence against this hypothesis. In particular, the lattice parameter for their sample is larger than ours, as shown in Figure 2b, while the corresponding ADPs are much smaller. Therefore, the cage volume does not appear to be the determining factor for the displacement parameter of the Ba atom at the 6*d* site in Ba_8_Al_*x*_Ga_16–*x*_Ge_30_.

**Figure 7:**
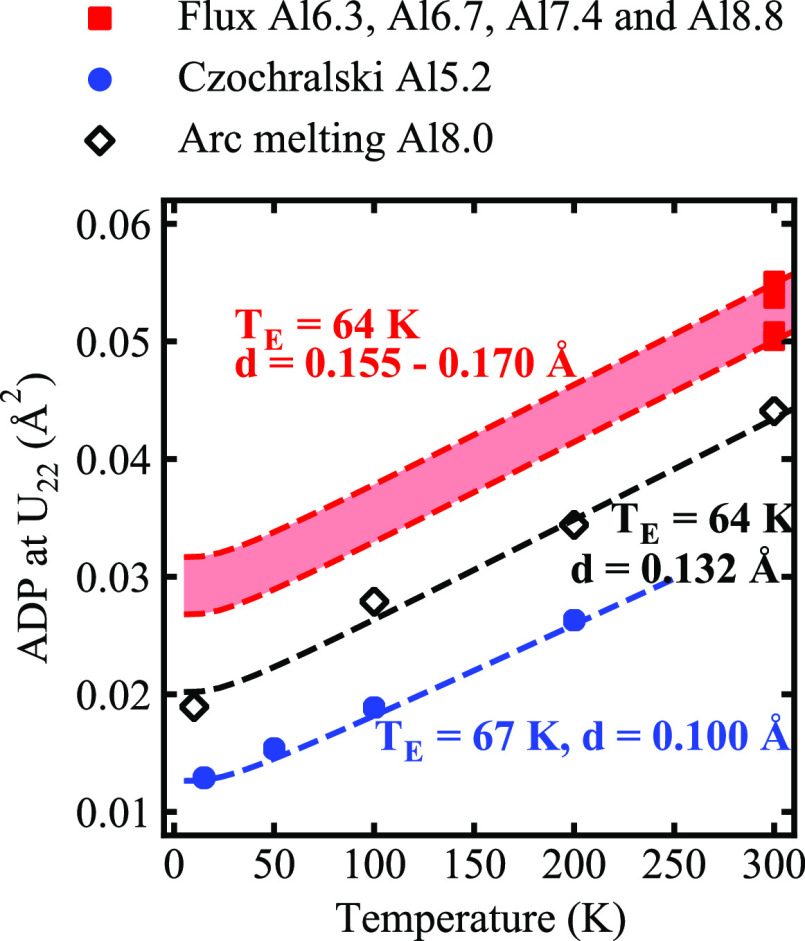
ADP (*U*_22_) of Ba at the 6*d* site (symbols), obtained from the refinement of neutron diffraction data, and the corresponding fits of the Einstein model (dashed lines (**− – –**)) for arc-melted samples^17^ (open black diamonds, ◇), Czochralski-pulled samples (solid blue circles, ●) and flux-grown samples (red squares, ■). Note that the latter are assumed to have the same Einstein temperature (*T*_E_ = 64 K). [Reproduced from ref (17). Copyright 2019, IEEE.]

In order to understand the different behaviors, we have used the Einstein expression to model the ADP in the *U*_22_ direction:^9,19^
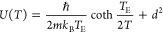
1The Einstein model (dashed lines) includes two fitting parameters: the Einstein temperature (*T*_E_), calculated from the slope of the curve, and temperature-independent disorder term (*d*), which corresponds to the intersection with the *y*-axis (Figure 7). Consequently, the Einstein temperature (*T*_E_) is calculated to be 67 and 64 K for the Czochralski and arc-melted samples, respectively. Note that *T*_E_ ranges from 59 K to 62 K for Ba_8_Ga_16_Ge_30_ and from 61 K to 69 K for Ba_8_Al_16_Ge_30_.^9,19^ Therefore, our estimates of the *T*_E_ for Ba_8_Al_*x*_Ga_16–*x*_Ge_30_ are very close to the values previously reported for Ba_8_Ga_16_Ge_30_ and Ba_8_Al_16_Ge_30_, which means that there is no obvious correlation between *T*_E_ and Al content. Under the assumption that the *T*_E_ values for the flux-grown and arc-melted samples are the same (*T*_E_ = 64 K), the temperature-independent disorder term (*d*) for the former is estimated to be between 0.155 Å and 0.170 Å, which is higher than the values obtained for the Czochralski and arc-melted samples. Therefore, the different ADPs observed for samples synthesized via different methods (Figure 7) are most likely the result of static disorder.

As noted in previous studies, the temperature-independent disorder term *d* represents the framework disorder in clathrates,^28^ because host element siting is different from one unit cell to the next in a real crystal. Therefore, the cages are asymmetric, which causes the Ba atoms to become displaced from the 6*d* site. Although a strong directional bonding between host and guest atoms would enhance *d*, this cannot explain the discrepancies between Ba_8_Al_*x*_Ga_16–*x*_Ge_30_ samples synthesized using different methods, since they are composed of the same elements. The similar *T*_E_ values also lend support to the conclusion that the bonding strength is indeed at the same level for all of the cases that we have considered. It is interesting to note that the SOFs vary the most at the 6*c* sites, which, as can be seen from Figure 1, form part of the six-rings in the large tetrakaidecahedral cages that surround the Ba atoms at the 6*d* site. The disorder term *d* extracted from the Einstein model, together with the chemical ordering analysis in the previous sections, lead to the conclusion that flux-grown samples are more disordered than the other samples, consistent with the outcome of the analysis of the SOFs. Apparently, the ADP of the guest atoms is related to the chemical ordering of the host sites. We have performed a detailed computational analysis of the local chemical environment for each of the Ba atoms occupying the 6*d* site (see the Supporting Information), with the hope to identify a clear correlation. Unfortunately, it was not possible to draw definitive conclusions. Therefore, we believe that this question deserves further study.

## Conclusions

We have studied quaternary type-I clathrates with the chemical composition Ba_8_Al_*x*_Ga_16–*x*_Ge_30_, synthesized by Czochralski and flux methods, using a combination of X-ray and neutron diffraction to determine the chemical ordering at the host sites. The experimental results obtained are in good agreement with atomic-scale simulations. However, the site occupations for the trivalent elements at the 6*c* site differ considerably, depending on the synthesis method. More precisely, the flux-grown samples show higher Ga occupation and lower Al occupation, compared to the Czochralski crystal. Since our computational approach takes the order–disorder transition into account, we have been able to show that, in particular, the SOFs for the flux-grown samples are consistent with the high-temperature disordered configuration. The experimental data for the Czochralski sample, on the other hand, lies between the theoretical predictions for the ground and high-temperature states, meaning that the Czochralski-grown sample is more ordered than the flux-grown samples. Furthermore, our results firmly establish that chemical ordering affects the ADPs for the Ba atoms at the 6*d* sites, since significantly higher ADPs are observed for the flux-grown samples. Therefore, this study further elucidates the impact of chemical ordering on physical properties, offering new perspectives for designing thermoelectric materials.

## Experimental Section

### Synthesis

Barium (crystalline dendritic solid, Alfa Aesar, 99.9%), gallium (metallic liquid, Sigma–Aldrich, 99.9995%), aluminum (beads, Sigma–Aldrich, 99.9%), and germanium (chips, Sigma–Aldrich, 99.999%) were used for the synthesis of quaternary Ba_8_Al_*x*_Ga_16–*x*_Ge_30_ single crystals via the Czochralski and the Ga-flux methods.^29,30^ Sample names “C-Al5.2” and “F-Alxx”, are used to refer to samples grown by the Czochralski and flux methods, respectively, where “5.2” and “xx” represent the number of Al atoms per unit cell, as determined from the structure refinement of XRD data.

To produce the C-Al5.2 sample, pure polycrystalline Ba_8_Ga_16_Ge_30_ and Ba_8_Al_16_Ge_30_ were first synthesized in accordance with a previously reported protocol.^31^ The as-synthesized Ba_8_Ga_16_Ge_30_ and Ba_8_Al_16_Ge_30_ were then mixed in a glassy carbon crucible and placed in a materials preparation and crystal growth system ((MPCGS)-Crystalox, Ltd.). The reaction chamber was flushed with argon four times before the temperature was increased to the melting point of the mixture and impurities were removed from the top of the melt. After the growth process was complete, the crystal was cooled to room temperature before being embedded in an epoxy polymer resin for further processing.

Meanwhile, the synthesis of each flux sample began with mixing of the pure elements in an alumina crucible in an argon-filled glovebox, with extra Ga added as the flux. The crucible was then sealed in an evacuated quartz tube and transferred to a vertical oven. It was subsequently heated to 1050 °C over a period of 17 h and kept there for 1 h, cooled to 970 °C in 4 h, and then slowly cooled to 955 °C within 100 h, before being rapidly cooled to room temperature. Single crystals were separated from the molten excess Ga with tweezers and then soaked in hot water, in order to wash away water-soluble impurities, as well as unreacted Ga. After that, the crystals were soaked in concentrated hydrochloric acid, then washed with ethanol and water and finally dried in air.

### Single-Crystal X-ray Diffraction

Single-crystal XRD data were collected using a Mo Kα radiation source (λ = 0.71073 Å). The single-crystal C-Al5.2 sample was measured on a Bruker D8 VENTURE diffractometer from 100 K to 300 K, while the flux grown samples were characterized at room temperature using an Oxford Diffraction Xcalibur3 diffractometer.

The structure was solved and refined using the Shelxl software.^32^ The space group for Ba_8_Al_*x*_Ga_16–*x*_Ge_30_ was found to be *Pm*3̅*n*. Since Ga and Ge have similar X-ray scattering cross sections, their SOFs were not refined. Ba is located at the 2*a* and 6*d* Wyckoff positions, while Al and Ga/Ge share the 6*c*, 16*i*, and 24*k* sites. All host sites (6*c*, 16*i*, and 24*k*) and the guest site 2*a* are assumed to be 100% occupied with isotropic ADPs. The guest atom at the 6*d* site can be described by an on-center or an off-center model. The on-center model means that the guest atom is located at the 6*d* site, which corresponds to the cage center, with a large anisotropic ADP. The off-center model means that the guest atom is positioned away from the cage center and split into four 24*k* sites. Each such site has an isotropic ADP but only 25% occupation.

### Single-Crystal Neutron Diffraction

Single-crystal neutron diffraction was performed on sample C-Al5.2 at the Extreme Environment Single Crystal Neutron Diffractometer (BL18 SENJU) at the Japan Proton Accelerator Research Complex (J-PARC).^33^ After cutting the sample into a rectangular shape with the approximate dimensions of 2.9 mm × 2.0 mm × 3.5 mm, diffraction data were, specifically, collected at temperatures of 15, 50, 100, and 200 K.

STARGazer was used for data processing, including the conversion of raw time-of-flight data, peak search and indexing, refinement of UB matrix, intensity correction of the wavelength dependence of incident neutrons, as well as the position dependence of detector efficiency, and the integration of Bragg reflections.^34^ Although the measured *d*-spacing extended to 0.4 Å, the minimum value that can be used in the analysis is 0.8 Å due to the strong extinction effect.

The neutron data was refined using the Jana2006^35^ software, with the initial model imported from the single-crystal XRD result. The Al SOFs were fixed, while Ga and Ge were separated in order to obtain the host atom distribution. Initially, the refinement was performed without any chemical constraints, but it turned out to have 17 Ga atoms and 23 Ge atoms per unit cell. Considering the fact that the difference in the scattering length, for neutrons, between Ga and Ge is only ∼10% (7.288 fm and 8.185 fm), a chemical constraint was applied that assumes a total of 30 Ge atoms in the unit cell. Finally, the extinction effect was refined.

### Powder Neutron Diffraction

The flux-grown samples were characterized by powder neutron diffraction using the Special Environment Powder Diffractometer (BL09 SPICA) at the Japan Proton Accelerator Research Complex (J-PARC). Approximately 3 g of each sample was ground to a fine powder, placed in a vanadium sample holder, and measured at 300 K.

Z-Rietveld was used for structure refinement. The initial model was imported from the single-crystal XRD result. The SOFs of Al were kept fixed, while Ga and Ge were separated in order to obtain the host atom distribution. A chemical constraint was applied that assumes a total of 30 Ge atoms in the unit cell.

### X-ray Fluorescence Analysis

The elemental composition of each sample was determined by XRF spectrometry (Axios Fast, Malvern PANalytical Ltd.), after having been ground to a powder, mixed with a binder, and finally pressed into the form of a 40-mm-wide pellet. Commercial standards (Omnian, Malvern PANalytical Ltd.) were used for calibration. It is challenging to accurately determine the content of Al in Ba_8_Al_*x*_Ga_16–*x*_Ge_30_.^11^ As shown in the XRF spectra in the Supporting Information, the intensity for Al Kα is only 0.64, which is at least 2 orders of magnitude lower than the signals from Ba, Ga, and Ge; therefore, we cannot calibrate the Al content using the commercial standard. Although we are able to determine the composition of Ba_8_Ga_16_Ge_30_ by XRF, the results are inconclusive for the samples that contain Al in the unit cell.

## Calculations

A combination of DFT, alloy CEs, MC, and Wang–Landau (WL) simulations were used to determine the most thermodynamically stable atomic configurations at temperatures between 0 K and 1200 K. The structures thus obtained were then used to predict the variations of the SOFs with both composition and temperature.

### Density Functional Theory

DFT calculations, based on the projector augmented wave method,^36,37^ were performed using the Vienna ab initio simulation package (VASP).^38^ The van der Waals density functional method^39^ with consistent exchange (vdW-DF-cx),^40^ as implemented in VASP, was used to take exchange-correlation effects into account. In total, 528 randomly generated Ba_8_Al_*x*_Ga_*y*_Ge_46–*x*–*y*_ structures, which included 132 Ba_8_Al_*x*_Ge_46–*x*_ and 132 Ba_8_Ga_*x*_Ge_46–*x*_ configurations, were relaxed, both in terms of the cell metrics and ionic positions, with Γ-centered 3 × 3 × 3 *k*-point meshes until the residual forces and absolute stresses were below 5 meV Å^–1^ and 0.1 kbar, respectively. In addition, Gaussian smearing, with a width of 0.1 eV, and a plane wave cutoff of 319 eV were employed for all calculations.

### Cluster Expansions

The relaxed structures were used to train alloy CEs with help of functionalities from the icet software package.^41^ As a first step, a cluster space was constructed from a prototype structure. A 5.4 Å cutoff, which is slightly smaller than half the unit-cell length of the primitive structure (10.99 Å), was applied for pairs as well as triplets, leading to 215 symmetry inequivalent clusters, including 6 singlets, 46 pairs, and 162 triplets. To estimate the quality of the CEs, cross-validation (CV) scores were calculated, using 90% of the available structures for training and the rest for validation, based on three different fitting methods, namely, least absolute shrinkage and selection operator (LASSO) and automatic relevance detection regression (ARDR), as well as ordinary least-squares (OLS) with recursive feature elimination (RFE). As has been reported elsewhere,^41,42^ the latter method gave consistently better results, both in terms of the root-mean-square error (RMSE) and the sparsity, and it was therefore used to construct the final CE. After fitting, the number of nonzero parameters had been reduced to 35, 23 of which corresponded to pairs and just 5 corresponded to triplets. Even so, the RMSE score for the “final model” was only 1.49 meV site^–1^, while the difference between the predicted energies and the reference DFT calculations were mostly scattered between ±2 meV atom^–1^, with only a handful of the 542 data points falling outside this interval.

### Monte Carlo Simulations

To predict the SOFs, the final CEs were sampled via ensemble-based WL and MC simulations, using 2 × 2 × 2 supercells, with help of the mchammer module in icet. Specifically, we performed two sets of ensemble-based MC simulations, the first of which was based on a canonical ensemble that spanned the entire composition range (6 ≤ *x* ≤ 20) and involved reducing the temperature from 1200 K to 0 K at a rate of 100 K per 22 000 MC cycles. Since each cycle consists of as many trial steps as there are atoms in the system (432), this corresponds to more than 12 × 10^7^ individual trial steps. The second case included equally as many steps but was performed using a hybrid approach, which consisted of randomly alternating between a canonical and a variance constrained semigrand canonical (VCSGC) ensemble.^43^ In particular, the former can swap the species on any two sites, while the latter is only allowed to switch the occupation on a single site from Ga to Al, or vice versa. This makes it possible to continuously vary the chemical composition, which is the advent of the VCSGC ensemble, while, at the same time keeping the number of trivalent atoms fixed and, thereby, ensuring that the entire range of interest is actually covered. Meanwhile, WL simulations were only performed for Ba_8_Al_*x*_Ga_16–*x*_Ge_30_ structures with 4, 6, or 8 Al atoms per unit cell. The sampling of the test models, on the other hand, was performed by running MC simulations at a fixed temperature (700 K) for the stoichiometric compositions (*x* = 16).

## Supporting Information Available

Supplemental figures (diffraction pattern for sample F-Al6.3; comparison between neutron and X-ray diffraction; SOFs versus temperature and composition; guest–host bond distances; SOFs and static displacements for all large cages; PESs and MSDs for Ba at the 6*d* site; characterization of four samples taken from the Czochralski-grown singe crystal; and XRF spectra of Czochralski-grown sample); supplemental tables (single-crystal X-ray diffraction data; single-crystal neutron diffraction data; powder neutron diffraction data); and supplementary notes (in-depth analysis of Ba displacements and local environments; characterization of single crystal grown via the Czochralski method)

## Accession Codes

CCDC 2096177, 2096181, 2096187, 2096192, 2096194, 2096199, 2096201, 2096204, 2096210, 2096211, 2096213, and 2096215 contain the supplementary crystallographic data for this paper. These data can be obtained free of charge via www.ccdc.cam.ac.uk/data_request/cif, or by emailing data_request@ccdc.cam.ac.uk, or by contacting The Cambridge Crystallographic Data Centre, 12 Union Road, Cambridge CB2 1EZ, UK; fax: +44 1223 336033.
