# Supplementary material for: Investigating the Chemical Ordering in Quaternary Clathrate Ba8AlxGa16–xGe30
Source: Inorg Chem. 2021 Nov 3;60(22):16977–85. doi: 10.1021/acs.inorgchem.1c01932 (PMC8596372; doi:10.1021/acs.inorgchem.1c01932)
Supplement: Supplementary file 1 — ic1c01932_si_001.pdf [file ic1c01932_si_001.pdf]

Supporting Information

Investigating the Chemical Ordering in Quaternary Clathrate $Ba_8Al_xGa_{16-x}Ge_{30}$

Yifei Zhang[1], Joakim Brorsson[1], Takashi Kamiyama[2], Takashi Saito[2,3], Paul Erhart[*,4] and Anders E. C. Palmqvist[*,1]

[1]Department of Chemistry and Chemical Engineering, Chalmers University of Technology, 41296 Gothenburg, Sweden
[2]Institute of Materials Structure Science, KEK, Tokai, Ibaraki, Japan
[3]SOKENDAI (The Graduate University for Advanced Studies), Tokai-mura, Naka-gun, Ibaraki 319-1106, Japan
[4]Department of Physics, Chalmers University of Technology, 41296 Gothenburg, Sweden

Email: erhart@chalmers.se; anders.palmqvist@chalmers.se

Contents

Supplementary Figures **S2**
S1. Diffraction pattern for sample F-Al6.3 . . . S2
S2. Comparison between neutron and X-ray diffraction . . . S3
S3. SOFs versus temperature and composition . . . S4
S4. Guest-host bond distances . . . S5
S5. SOFs and static displacements for all large cages . . . S6
S6. PESs and MSDs for Ba at the 6*d* site . . . S7
S7. Characterization of four samples taken from the Czochralski-grown singe crystal . . . S8
S8. XRF spectra of Czochralski-grown sample . . . S9

Supplementary Tables **S10**
S1. Single crystal X-ray diffraction data . . . S10
S2. Single crystal neutron diffraction data . . . S11
S3. Powder neutron diffraction data . . . S12

Supplementary Notes **S13**
S1. In-depth analysis of Ba displacements and local environments . . . S13
S2. Characterization of single crystal grown by Czochralski method . . . S13

Supplementary References **S15**

Supplementary Figures

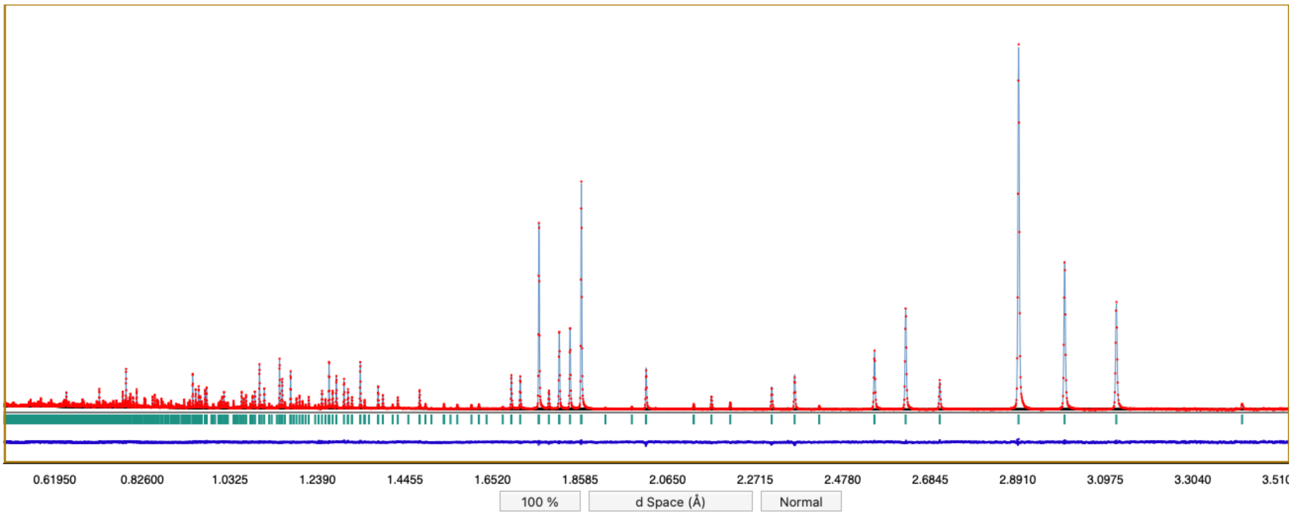

Supplementary Figure S1: Diffraction pattern for sample F-Al6.3. Powder neutron diffraction data for sample F-Al6.3, which includes the observed (red dots) and calculated (light blue curve) patterns, the difference (dark blue curve) as well as the expected Bragg peak positions (green bars).

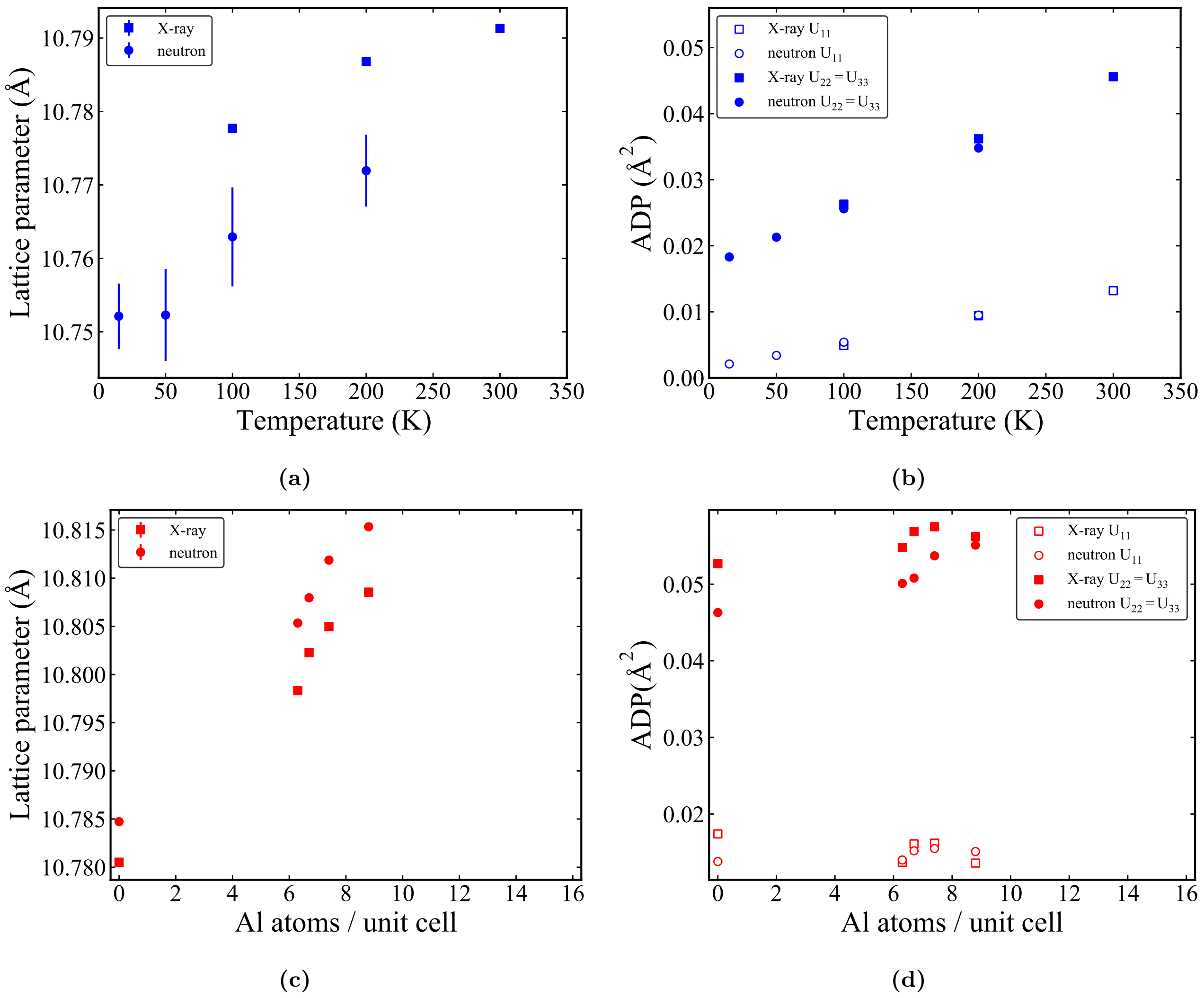

Supplementary Figure S2: Comparison between neutron and X-ray diffraction. Comparison between the structure refinement results for neutron and X-ray diffraction, which includes lattice parameters (a, c) and atomic displacement parameters (ADPs) for the Ba atoms at the 6*d* sites (b, d) for the Czochralski (a, b) and flux-grown (c, d) samples.

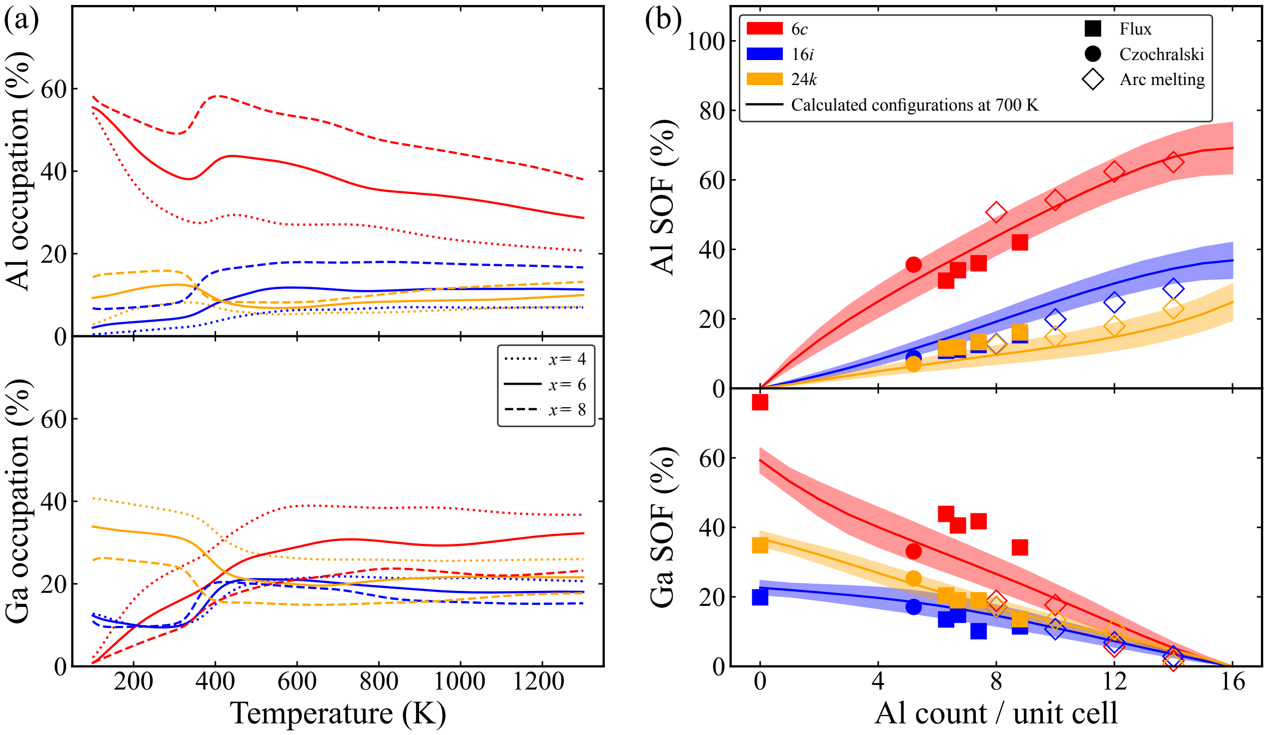

Supplementary Figure S3: SOFs versus temperature and composition. Calculated Al and Ga occupations for $Ba_8Al_xGa_{16-x}Ge_{30}$ at the 6*c* (red), 16*i* (blue) and 24*k* (orange) sites. This includes (a) the temperature variations for $x = 4, 6, 8$, extracted from Wang-Landau (WL) simulations, and (b) mean values (solid lines) and standard deviations (filled curves) as functions of the composition, at 700 K. The latter data has, specifically, been obtained by sampling 100 test models, constructed using a Bayesian approach (1, 2), via the Monte Carlo (MC) method. Comparable experimental data for flux-grown (filled squares), Czochralski-pulled (filled circles) and reference arc-melted (open diamonds) samples is also shown.

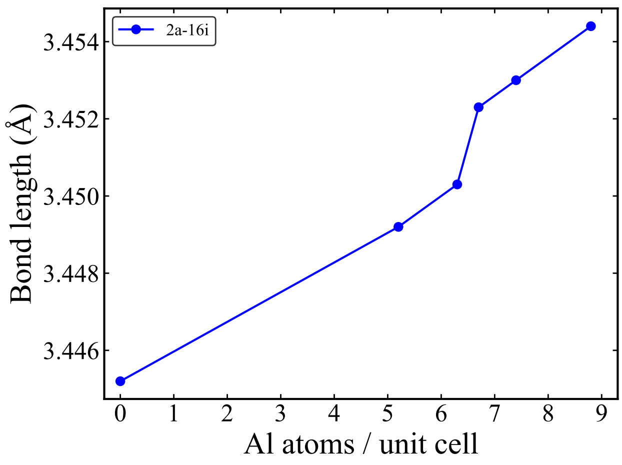

(a)

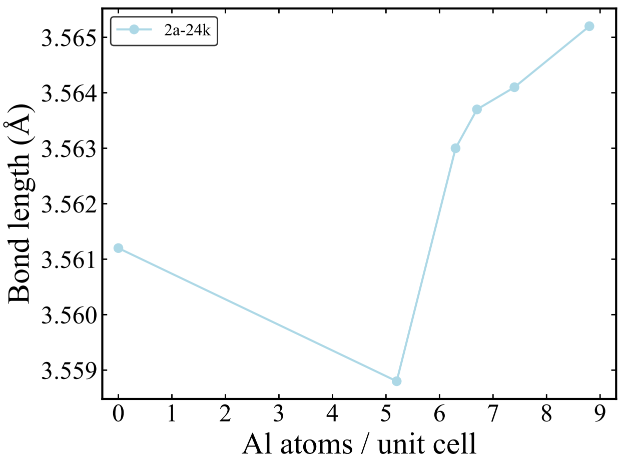

(b)

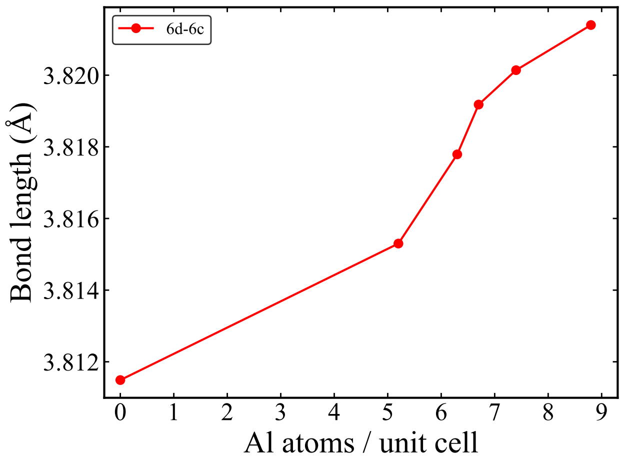

(c)

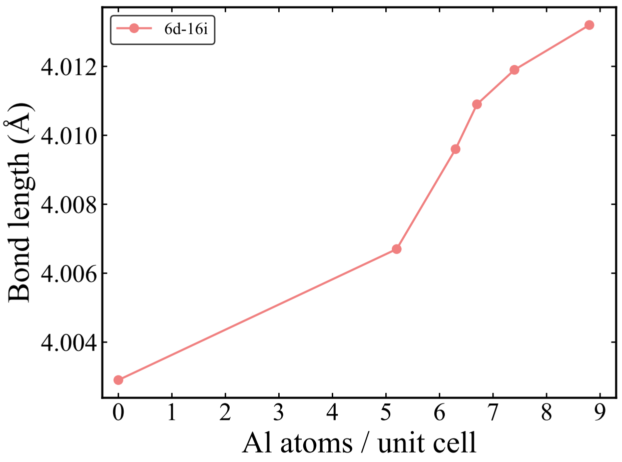

(d)

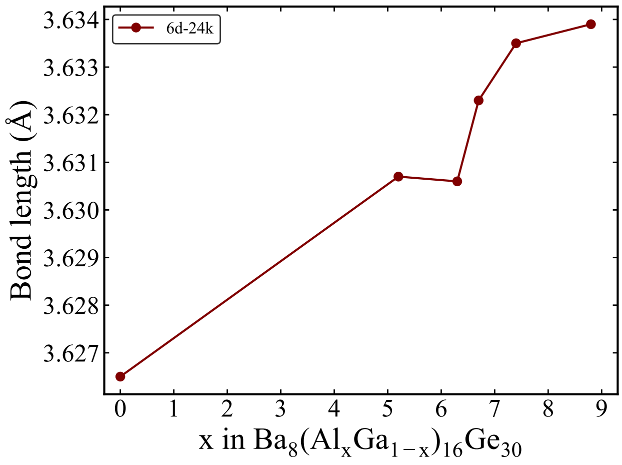

(e)

Supplementary Figure S4: Guest-host bond distances. Bond distances between guests and host atoms on different crystallographic positions: (a) $2a$ and $16i$, (b) $2a$ and $24k$, (c) $6d$ and $6c$, (d) $6d$ and $16i$, as well as (e) $6d$ and $24k$.

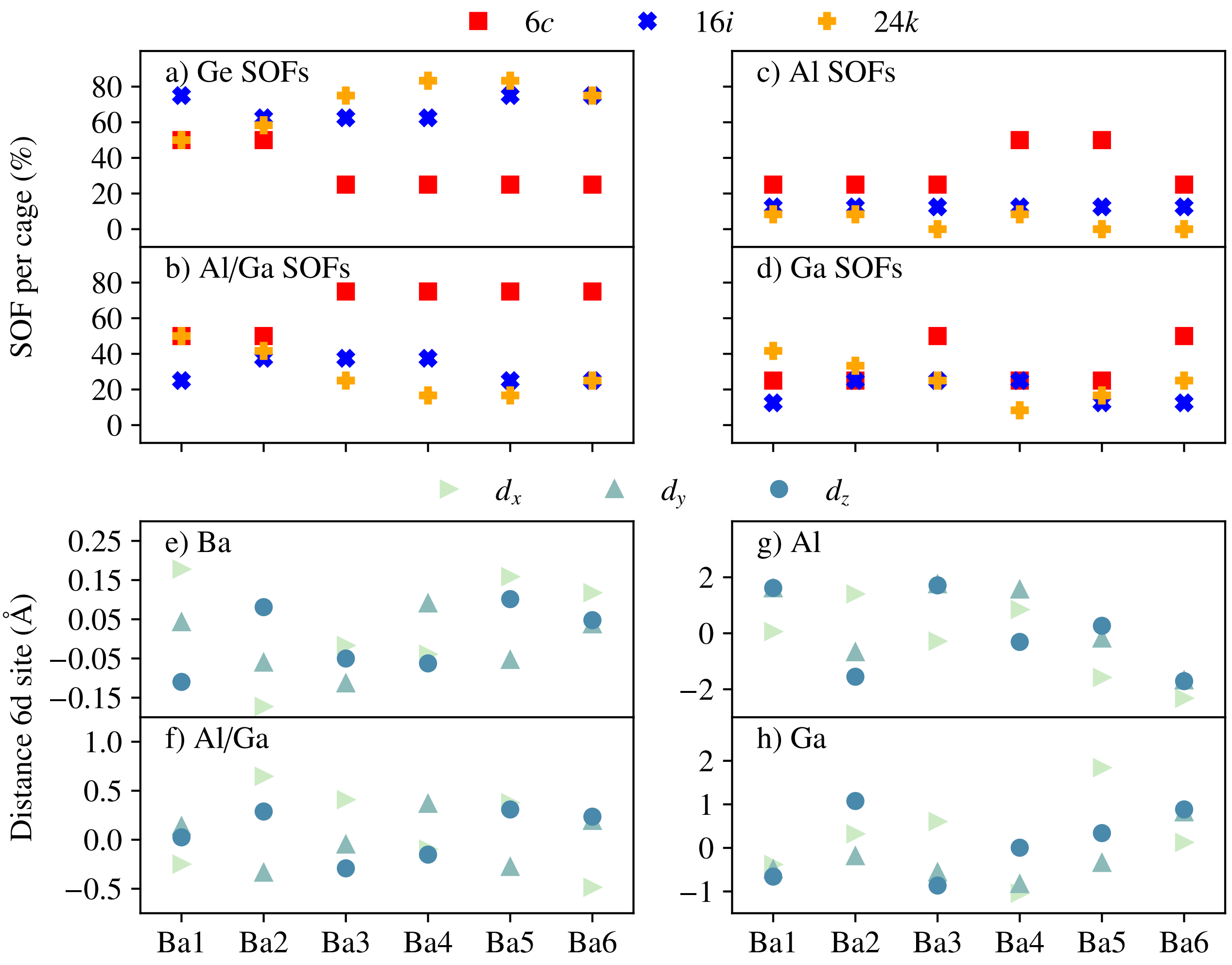

Supplementary Figure S5: SOFs and static displacements for all large cages. Site occupation factors (SOFs) for (a) Ge, (b) Al/Ga, (c) Al, and (d) Ga at the 6*c* (red square), 16*i* (blue cross) and 24*k* (orange plus) sites in the large (tetrakaidecahedral) cages. Also shown are (e) the static displacements of the Ba atoms from the ideal (6*d*) position as well as the distance from the latter to the (f) Al/Ga, (g) Al, and (h) Ga centroids.

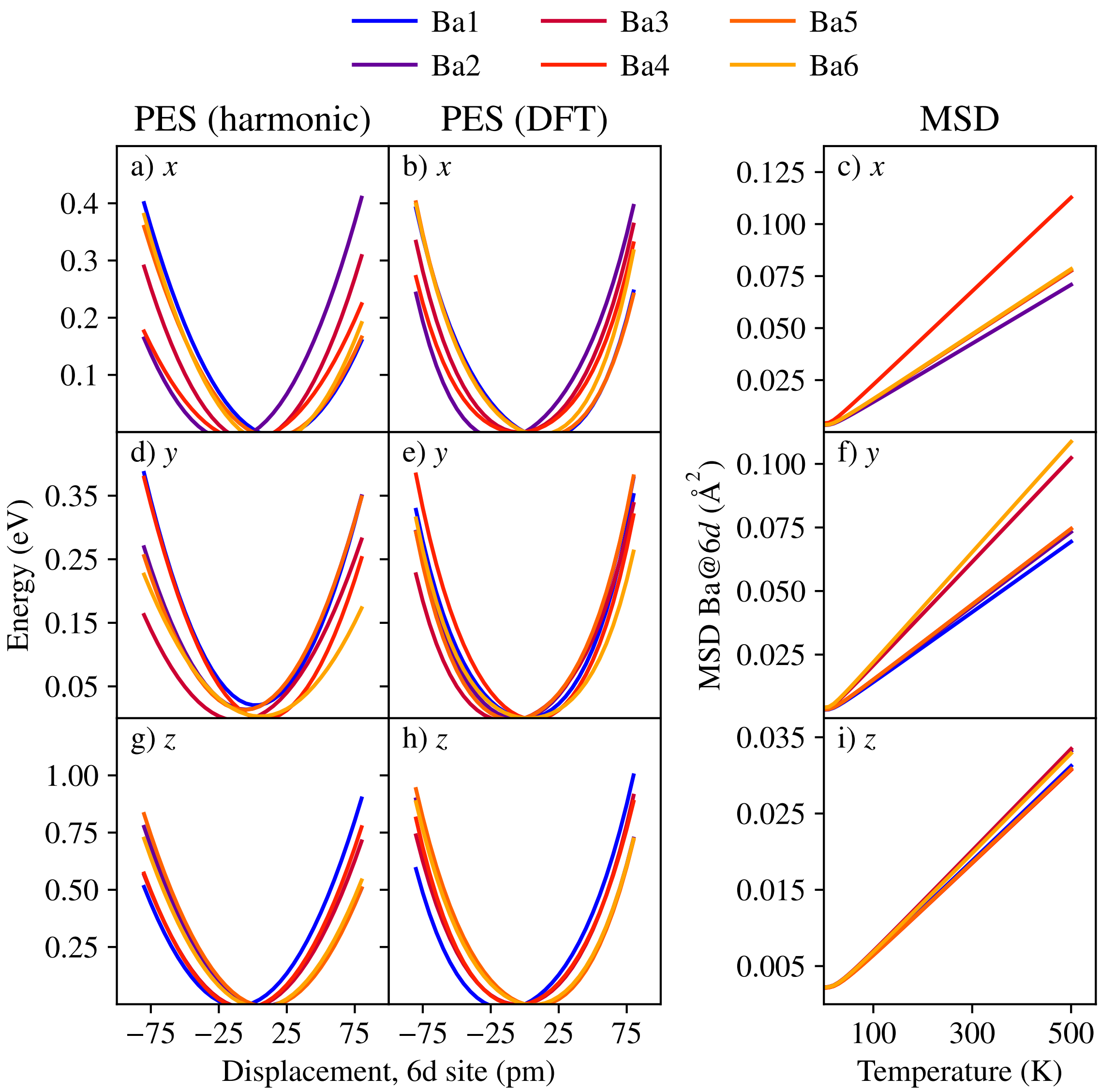

Supplementary Figure S6: PESs and MSDs for Ba at the $6d$ site. Potential energy surfaces (PESs) obtained using the harmonic approximation (a,d,g) and directly extracted from density functional theory (DFT) calculations (b,e,h) as well as mean square displacements (MSDs) (c,f,i) for each of the Ba atoms located at $6d$ sites. Specifically, both properties have been evaluated along the local x (a,b,c), y (d,e,f), and z (g,h,i) axes, which are set to be parallel to the two two-fold and single four-fold rotation axes, in accordance with the convention used by, e.g., Takasu *et al.* (3).

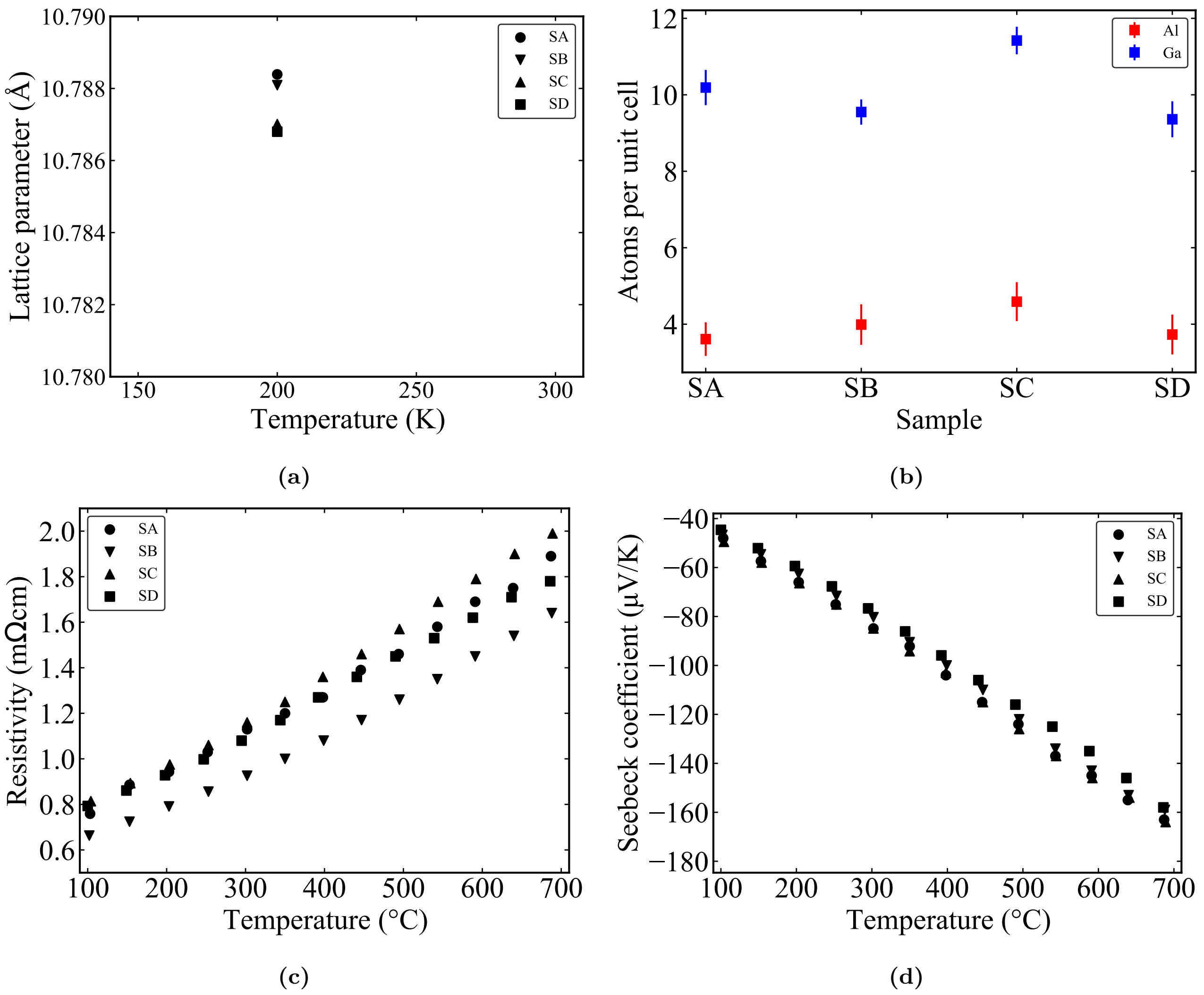

Supplementary Figure S7: Characterization of four samples taken from the Czochralski-grown singe crystal. (a) Lattice parameter of the four samples, obtained from the single crystal X-ray diffraction at 200 K. (b) Al and Ga content of the four samples. Composition was determined from EDX and then normalized to 8 Ba atoms per unit cell. (c) and (d) Resistivity and Seebeck coefficient of these four samples, measured by ZEM3 instrument.

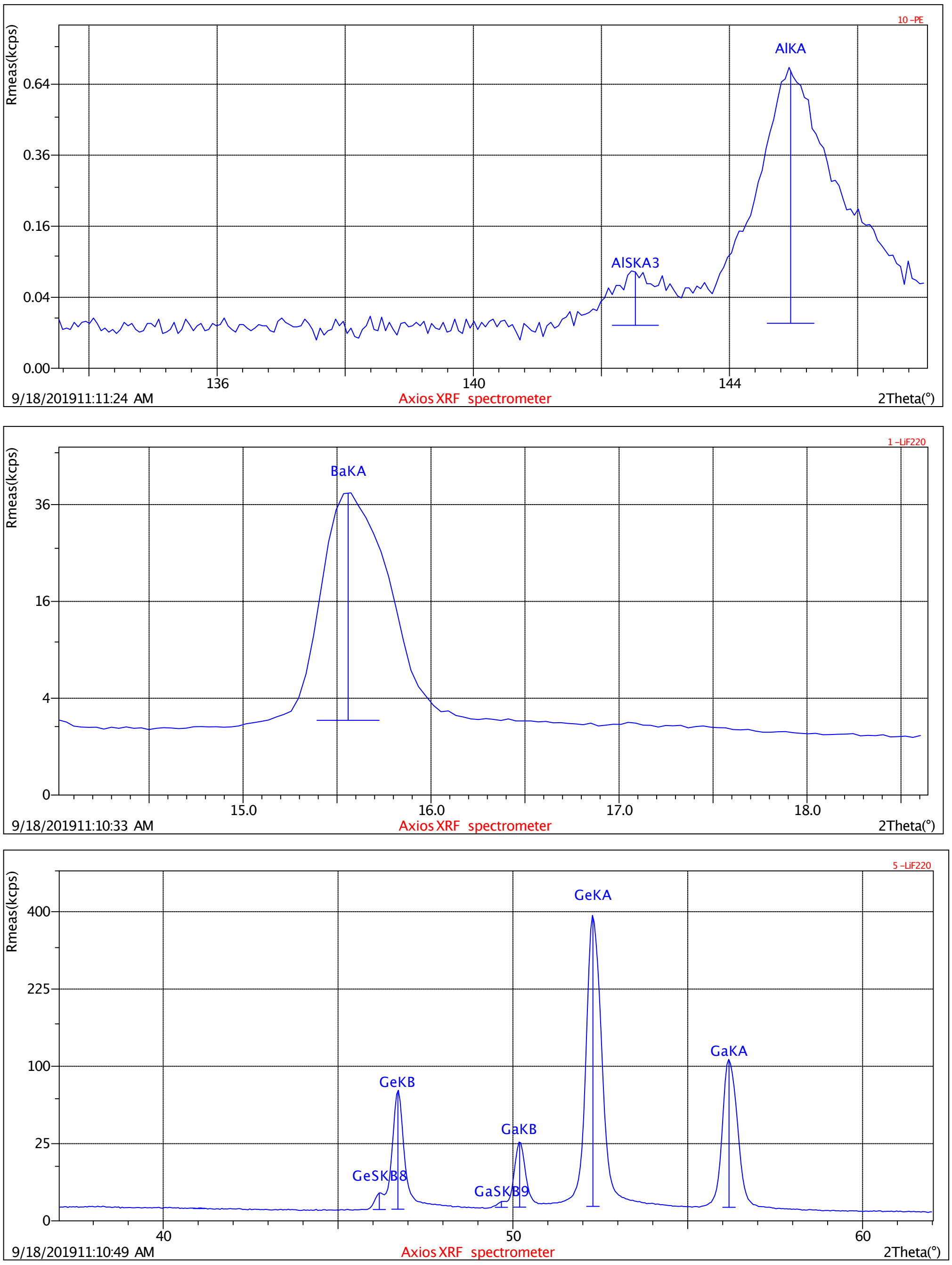

Supplementary Figure S8: XRF spectra of Czochralski-grown sample. The intensity for the Al, Ba, Ga and Ge Kα characteristic lines are shown.

Supplementary Tables

Supplementary Table S1: Single crystal X-ray diffraction data. Selected crystallographic information from single crystal X-ray diffraction data at 300 K.

sample	F-Al0.0	C-Al5.2	F-Al6.3	F-Al6.7	F-Al7.4	F-Al8.8
experiment method	single crystal X-ray, $\lambda = 0.71073$ Å					
space group	$Pm\bar{3}n$					
model	Ba 6*d* aniso					
lattice parameter (Å)	10.78052(9)	10.7913(5)	10.79833(11)	10.80228(8)	10.80498(6)	10.80855(9)
number of reflections	24148	37973	24705	24385	24833	24629
$N/N(I > 2\sigma(I))$	410 / 391	575 / 555	410 / 393	415 / 397	415/403	415/412
μ (mm^{-1})	33.257	30.881	30.821	30.787	30.764	30.734
$N_{\text{parameter}}/N_{\text{constraints}}$	14 / 0	17 / 3	17 / 3	17 / 3	17 / 3	17 / 3
$\theta_{\max}$ (°)	31.831	36.287	31.634	31.898	31.889	31.877
$R_F/wR_F(I > 2\sigma(I))$	0.0398 / 0.0960	0.0264 / 0.0681	0.0286 / 0.0714	0.0318/0.0677	0.0312/0.0700	0.0322/0.0716
Goodness-of-fit (GOF)	1.331	1.034	1.203	1.229	1.236	1.317
U_{11} (Ba 6*d*) (Å^2)	0.0174(6)	0.0132(3)	0.0137(5)	0.0161(5)	0.0162(5)	0.0136(5)
$U_{22} = U_{33}$ (Ba 6*d*) (Å^2)	0.0527(7)	0.0456(4)	0.0548(6)	0.0569(6)	0.0575(6)	0.0562(6)
U_{iso} (Ba 2*a*) (Å^2)	0.0114(4)	0.00758(19)	0.0081(3)	0.0103(3)	0.0101(3)	0.0080(3)
U_{iso} (host 6*c*) (Å^2)	0.0113(5)	0.0065(3)	0.0075(5)	0.0096(5)	0.0092(5)	0.0068(6)
U_{iso} (host 16*i*) (Å^2)	0.0093(4)	0.00620(18)	0.0066(3)	0.0088(3)	0.0085(3)	0.0062(3)
U_{iso} (host 24*k*) (Å^2)	0.0103(4)	0.00630(15)	0.0070(3)	0.0090(2)	0.0087(3)	0.0064(3)
SOF(Al, 6*c*) (%)	/	35.0(19)	31(2)	34(2)	36(2)	42(2)
SOF(Al, 16*i*) (%)	/	8.7(17)	10.8(19)	11.1(18)	12.5(18)	15.3(19)
SOF(Al, 24*k*) (%)	/	7.7(17)	11.5(18)	11.9(18)	13.3(18)	16.1(19)
SOF(Ga/Ge, 6*c*) (%)	99.9(10)	65.0(12)	68.6(15)	66.3(14)	63.9(14)	58.2(16)
SOF(Ga/Ge, 16*i*) (%)	97.6(6)	91.2(9)	89.1(11)	88.7(11)	87.4(11)	84.5(12)
SOF(Ga/Ge, 24*k*) (%)	98.3(7)	92.1(9)	88.4(11)	88.0(10)86.7(10)	83.9(11)	

Supplementary Table S2: Single crystal neutron diffraction data. Selected crystallographic information from single crystal neutron diffraction data for sample C-Al5.2.

sample	C-Al5.2			
experiment method	single crystal neutron, time of flight			
space group	$Pm\bar{3}n$			
model	Ba 6d aniso			
temperature (K)	15	50	100	200
lattice parameter (Å)	10.75260(10)	10.75450(10)	10.76290(10)	10.77090(10)
$N/N(I > 3\sigma(I))$	13207 / 9211	13097/9010	13154/8827	13133/8199
extinction method	isotropic, Gaussian type 1			
extinction coefficient	990(9)	935(9)	915(9)	930(9)
$N_{\text{parameter}}/N_{\text{restraints}}$	26 / 16			
θ_{max}	82.41	80.95	81.76	82.64
$R_F/wR_F(I > 3\sigma(I))$	0.0509/0.1153	0.0510/0.1176	0.0519/0.1164	0.0555 / 0.1198
GOF	2.51	2.55	2.41	2.35
U_{11} (Ba 6d) (Å^2)	0.0021(2)	0.0034(2)	0.0054(3)	0.0095(3)
$U_{22} = U_{33}$ (Ba 6d) (Å^2)	0.0183(2)	0.0213(2)	0.0256(3)	0.0348(3)
U_{iso} (Ba 2a) (Å^2)	0.00097(13)	0.00155(13)	0.00236(13)	0.00500(16)
U_{iso} (host 6c) (Å^2)	0.00095(9)	0.00134(9)	0.00203(9)	0.00419(11)
U_{iso} (host 16i) (Å^2)	0.00130(4)	0.00166(4)	0.00243(4)	0.00456(5)
U_{iso} (host 24k) (Å^2)	0.00135(3)	0.00163(4)	0.00244(4)	0.00458(4)
SOF(Al, 6c) (%)	35.6			
SOF(Al, 16i) (%)	8.7			
SOF(Al, 24k) (%)	7			
SOF(Ga, 6c) (%)	33.1(16)			
SOF(Ga, 16i) (%)	17.1(10)			
SOF(Ga, 24k) (%)	25.3(5)			
SOF(Ge, 6c) (%)	31.3(16)			
SOF(Ge, 16i) (%)	74.2(10)			
SOF(Ge, 24k) (%)	67.7(5)			

Supplementary Table S3: Powder neutron diffraction data. Selected crystallographic information from powder neutron diffraction data at 300 K.

sample	F-Al0.0	F-Al6.3	F-Al6.7	F-Al7.4	F-Al8.8
experiment method	powder neutron, time of flight				
lattice parameter (Å)	10.78473(3)	10.805345(4)	10.807967(4)	10.811873(5)	10.815336(3)
R_{wp}/wR_F (%)	5.75 / 4.96	3.97 / 4.5	3.95/5.01	4.43/3.53	2.93/4.25
S	2.03	2.15	2.26	2.14	2.88
U_{11} (Ba 6d) (Å^2)	0.01380	0.0140(4)	0.0152(4)	0.0155(5)	0.0151(3)
$U_{22} = U_{33}$ (Ba 6d) (Å^2)	0.04630	0.0501(3)	0.0508(4)	0.0537(4)	0.0551(3)
U_{iso} (Ba 2a) (Å^2)	0.00770	0.0078(2)	0.0082(2)	0.0089(3)	0.01055(16)
U_{iso} (host 6c) (Å^2)	0.00664	0.00658(12)	0.00701(13)	0.00700(16)	0.00859(13)
U_{iso} (host 16i) (Å^2)	0.00734	0.00725(6)	0.00753(6)	0.00733(8)	0.00869(5)
U_{iso} (host 24k) (Å^2)	0.00688	0.00704(6)	0.00719(6)	0.00706(7)	0.00828(5)
SOF(Al, 6c) (%)	/	31	34	36	42
SOF(Al, 16i) (%)	/	10.8	11.1	12.5	15.3
SOF(Al, 24k) (%)	/	11.5	11.9	13.3	16.1
SOF(Ga, 6c) (%)	71.0(18)	40.3(11)	33.5(12)	32.0(13)	28.8(14)
SOF(Ga, 16i) (%)	15.3(10)	10.9(6)	12.0(6)	8.3(7)	7.0(7)
SOF(Ga, 24k) (%)	39.1(6)	23.0(5)	22.7(4)	22.7(5)17.9(4)	
SOF(Ge, 6c) (%)	29.0(18)	28.7(11)	32.5(12)	32.0(13)	29.2(14)
SOF(Ge, 16i) (%)	84.7(10)	78.3(6)	76.9(6)	79.2(7)	77.7(7)
SOF(Ge, 24k) (%)	60.9(6)	65.5(5)	65.4(4)	64.0(5)	66.0(4)

Supplementary Notes

Supplementary Note S1: In-depth analysis of Ba displacements and local environments.

In order to elucidate the origin of the difference in the ADPs displayed by the $Ba_8Al_xGa_{16-x}Ge_{30}$ samples synthesized using the Czochralski and Ga-flux methods (see Figure 5), we have carried out an in-depth investigation of the local chemical environment for each of the six Ba atoms that reside within the large (tetrakaidecahedral) cages, per unit cell. Specifically, we have considered the configuration ($x = 5$) that best corresponds to the average cluster vector sampled at 700 K. After relaxing this structure, using the procedure described in the "Calculations" section, the SOFs for all host sites that form part of the cage were extracted (Figure S5a-d). In addition, we calculated the static displacements of the Ba atoms relative to the ideal, 6*d*, Wyckoff site (Figure S5e). In order to get an estimate of the asymmetry of the distribution of the host species, we also determined the distances from the Ba positions to the centroids, i.e. the geometrical centers, of the Al, Ga, and Al/Ga atoms (Figure S5f-i). Note that all displacements have been evaluated along the local symmetry axes, which were chosen so as to agree with the convention used by, for instance, Takasu *et al.* (3). This means that the z direction is set parallel to the four-fold rotation axis while x and y match the two, perpendicular, two-fold axes, all of which pass through the 6*d* site located at the cage center.

Our analysis of the data described above indicates that the situation is relatively complicated since we are unable to find a distinctive one-to-one correlation between the Ba displacements and either the asymmetry or the SOFs. Even so, it is possible to distinguish some interesting trends. For instance, the MSDs along the x and y directions are exceptionally large for Ba1 and Ba2, which are the only Ba atoms found in cages where the Al/Ga occupation at the 6*c* site is 50 %. Regardless of which of the four available 6*c* sites that the Al and Ga atoms occupy, this should lead to a pronounced asymmetry, even though the centroid deviates only slightly from the cage center. This result agrees with the observation by Takasu *et al.* (3) that the Ba atoms in $Ba_8Ga_{16}Ge_{30}$ tend to be displaced towards the 6*c* position. It can, therefore, be concluded that there exists some degree of correlations between static displacement and Al/Ga SOF at the latter site. In addition, if one compares the deviations of the Ba atom and Al/Ga centroid from the cage centers for the other cases (Ba3-6), it is clear that the variations are very similar.

To gain further information regarding the relationship between the local chemical ordering and the guest atoms behavior, we have also calculated the MSDs and PESs (Figure S6) for the Ba atoms at the 6*d* site. Specifically, the former were determined using harmonic force constants (FCs) extracted from DFT caluclation using the HIPHIVE software package (4). This was achieved by first generating 10 "rattled" structures, which involves displacing all atoms in the unit cell based on a Gaussian distribution, with a standard deviation of 0.02 Å. Next, we fitted a second order FC potential, by employing the ordinary least squares (OLS) method, to the sets of forces, which were calculated using Vienna ab initio simulation package (VASP), and displacements were thus obtained. Finally, we used the FCs extracted from this potential to calculate the harmonic MSDs (Figure S6c,f,i) with the help of PHONOPY (5). The PESs, meanwhile, were obtained by computing the energies for a suitable collection of structures using both DFT (Figure S6a,d,g) and the harmonic FCs (Figure S6b,e,h). Each structure set was, more precisely, generated by separately displacing each of the individual Ba atoms, located at the 6*d* sites, along the three local symmetry axes in 0.05 Å increments relative to the ideal position.

While it is hard to draw any definitive conclusions based on the MSDs and PESs, these partly validate patterns we distinguished when analyzing the SOFs and static Ba displacements. In particular, it is evident that the MSDs for the Ba1 and Ba2 atoms show the weakest temperature dependence (Figure S6c,f,i) as well as the most asymmetric, and off-centered, PESs, especially along the x axis (Figure S6a). Still, one should emphasize that the local variations are substantial for both properties and, crucially, does not seem to be directly correlated with either the SOFs or the distances to the host centroids. Taken together, however, the combined results indicate that the, static, Ba displacements are affected by the asymmetry in the distribution of the host species in the surrounding cage and is especially sensitive to the occupation of the 6*c* site.

Supplementary Note S2: Characterization of single crystal grown by Czochralski method.

A 10-cm long single crystal was grown at a rate of $1\,\mathrm{cm\,h^{-1}}$ by Czochralski method. In order to check the homogeneity along the pulling direction, four samples were cut from the as-synthesized single crystal: SA (3 cm to the top), SB (4 cm to the top), SC (5 cm to the top) and SD (7 cm to the top). It should be noted that SD is sample C-Al5.2 in the main article, which was characterized by both single crystal X-ray and neutron diffraction.

The lattice parameter of these four samples, obtained from the single crystal X-ray diffraction, is consistent (Figure S7a). The variation between four samples is small, changing from 10.7868 to 10.7884 Å. These four

samples were characterized by SEM/EDX, and the composition was determined over 20 scanning points. As shown in Figure S7b, the obtained Al and Ga content of each sample is also quite consistent, meaning there is no compositional gradient along the growth direction. Samples were also measured by XRF (Figure S8), but the intensity of Al Kα line is too small compared to the other elements, so the Al content cannot be accurately determined by XRF. The electrical transport properties of four samples were measured (Figure S7c and d), and show excellent consistency between samples.

Based on these characterization, it is concluded that no obvious compositional gradient is observed along the growth direction, the composition for the Czochralski-grown single crystal is relatively homogeneous.

Supplementary References

[1] T. Mueller and G. Ceder, *Bayesian approach to cluster expansions,* Phys. Rev. B **80**, 024103 (2009). doi:10.1103/PhysRevB.80.024103.

[2] D. Foreman-Mackey, D. W. Hogg, D. Lang, and J. Goodman, *emcee: The MCMC Hammer,* Publications of the Astronomical Society of the Pacific **125**, 306 (2013). doi:10.1086/670067.

[3] Y. Takasu, T. Hasegawa, N. Ogita, M. Udagawa, M. A. Avila, K. Suekuni, and T. Takabatake, *Off-center rattling and cage vibration of the carrier-tuned type-I clathrate $Ba_8Ga_{16}Ge_{30}$ studied by Raman scattering,* Phys. Rev. B **82**, 134302 (2010). doi:10.1103/PhysRevB.82.134302.

[4] F. Eriksson, E. Fransson, and P. Erhart, *The Hiphive Package for the Extraction of High-Order Force Constants by Machine Learning,* Advanced Theory and Simulations **2**, 1800184 (2019). doi:10.1002/adts.201800184.

[5] A. Togo and I. Tanaka, *First principles phonon calculations in materials science,* Scripta Materialia **108**, 1 (2015). doi:https://doi.org/10.1016/j.scriptamat.2015.07.021.
